# Author Correction: An end-to-end pipeline for succinic acid production at an industrially relevant scale using *Issatchenkia orientalis*

**DOI:** 10.1038/s41467-024-45331-x

**Published:** 2024-02-07

**Authors:** Vinh G. Tran, Somesh Mishra, Sarang S. Bhagwat, Saman Shafaei, Yihui Shen, Jayne L. Allen, Benjamin A. Crosly, Shih-I Tan, Zia Fatma, Joshua D. Rabinowitz, Jeremy S. Guest, Vijay Singh, Huimin Zhao

**Affiliations:** 1 Department of Chemical and Biomolecular Engineering, University of Illinois at Urbana-Champaign, Urbana, IL 61801 USA; 2 Carl R. Woese Institute for Genomic Biology, University of Illinois Urbana-Champaign, Urbana, IL 61801 USA; 3 Department of Agricultural and Biological Engineering, University of Illinois at Urbana-Champaign, Urbana, IL 61801 USA; 4 Department of Civil and Environmental Engineering, University of Illinois Urbana-Champaign, Urbana, IL 61801 USA; 5 Department of Chemistry and Lewis Sigler Institute for Integrative Genomics, Princeton University, Princeton, NJ 08540 USA; 6 Departments of Chemistry, Biochemistry, and Bioengineering, University of Illinois at Urbana-Champaign, Urbana, IL 61801 USA

**Keywords:** Metabolic engineering, Metabolic engineering, Chemical engineering, Chemical engineering

Correction to: *Nature Communications* 10.1038/s41467-023-41616-9, published online 3 October 2023

The original version of this Article contained an error in Fig. 2, in which the legends for “titer” and “productivity” were swapped. This has been corrected in both the PDF and HTML versions of the Article.

