# Supplementary material for: An end-to-end pipeline for succinic acid production at an industrially relevant scale using Issatchenkia orientalis
Source: Nat Commun. 2023 Oct 3;14:6152. doi: 10.1038/s41467-023-41616-9 (PMC10547785; doi:10.1038/s41467-023-41616-9)
Supplement: Supplementary file 1 — Supplementary Information [file 41467_2023_41616_MOESM1_ESM.pdf]

An end-to-end pipeline for succinic acid production at an industrially relevant scale using *Issatchenkia orientalis*

Tran *et al.*

Supplementary Method 1. Plasmid construction

p102-MAE1. *SpMAE1* was codon optimized and synthesized by TWIST Bioscience (San Francisco, CA) and PCR-amplified using primers MAE1.F and MAE1.R. The amplified *SpMAE1* was inserted into *Sfo*I-digested p102 using HiFi DNA Assembly (New England Biolabs, Ipswich, MA).

p101a-PYC, p101a-MDH, p101a-FUMR, and p101a-FRD. *PYC* was PCR-amplified from p415-E using primers PYC.F and PYC.R. The amplified *PYC* was inserted into *Sfo*I-digested p101a using HiFi DNA Assembly. Construction of p101a-MDH, p101a-FUMR, and p101a-FRD was carried out similarly using the corresponding primer pairs.

p101a-DAK. *DAK* was PCR-amplified from genomic DNA of SD108 using primers DAK.F and DAK.R. The amplified *DAK* was inserted into *Sfo*I-digested p101a using HiFi DNA Assembly.

p102-PaGDH. *PaGDH* was codon optimized and synthesized by TWIST Bioscience and PCR-amplified using primers PaGDH.F and PaGDH.R. The amplified *PaGDH* was inserted into *Sfo*I-digested p102 using HiFi DNA Assembly.

p416-PaGDH-DAK. The cassette TDH3p-*DAK*-g3376t was PCR-amplified from plasmid p101a-DAK using primers p101a-DAK.F and p101a-DAK.R. The cassette g853p-*PaGDH*-g3767t was PCR-amplified from plasmid p102-PaGDH using primers p102-PaGDH.F and p102-PaGDH.F. Plasmid pRS416 was digested with *Xho*I and *Sac*I. The cassettes TDH3p-*DAK*-g3376t and g853p-*PaGDH*-g3767t and the digested pRS416 were used for DNA assembler[1] of pRS416-PaGDH-DAK.

p101a-SUC2. *SpMAE1* was codon optimized and synthesized by TWIST Bioscience and PCR-amplified using primers MAE1.F and MAE1.R. The amplified *SpMAE1* was inserted into *Sfo*I-digested p102 using HiFi DNA Assembly.

pVT36b-PDC, pVT36b-GPD, pVT36b-g3473, pVT36b-g3068, pVT36b-NDE, p36b-g3837, p36b-g1398, and p36b-g2945. The gBlocks PDC, GPD, g3473, g3068, NDE, g3837, g1398, and g2945 were inserted into pVT36b using Golden Gate Assembly[2] using *Bsa*I.

pVT36b-int1, pVT36b-int2, pVT36b-int3, and pVT36b-int5. Primers site1.spacer.R and site1.spacer.R were annealed using T4 Polynucleotide Kinase (New England Biolabs, Ipswich, MA), and the product was then inserted into pVT36b using Golden Gate Assembly with *Bsa*I to construct pVT36b.int1. Construction of pVT36b-int2, pVT36b-int3, and pVT36b-int5 was carried out similarly using the corresponding primer pairs.

Supplementary Method 2. Strain construction

Strain SA/MAE1. The cassette g853p-*SpMAE1*-g3767t was PCR-amplified from plasmid p102-MAE1 and co-transformed with pVT36b-int2 into *Issatchenkia orientalis* SD108. Yeast colonies were screened for integration of g853p-*SpMAE1*-g3767t cassette by PCR using primers MAE1.check.F and int2.down.R. Plasmid pVT36b-int2 was then cured using SC-FOA, leading to strain MAE1/ura3Δ.

Plasmid pRS416-SA-site1 was digested with *Mlu*I to liberate the reductive TCA cassette. The reductive TCA cassette was co-transformed with pVT36b-int1 into strain MAE1/ura3Δ. Yeast colonies were screened for integration of reductive TCA cassette by PCR using primers SA.check.F and int1.down.R. Plasmid pVT36b-int1 was then cured using SC-FOA, resulting in strain SA/MAE1/ura3Δ. Uracil prototrophy was restored by integration of URA3p-*URA3*-PDC1t cassette, amplified using primers URA3.F and URA3.R from p101a, to strain SA/MAE1/ura3Δ, leading to strain SA/MAE1.

*Strain SA/MAE1/pdc*Δ*/gpd*Δ. For *PDC* deletion, plasmid pVT36b-PDC was transformed into strain SA/MAE1/ura3Δ. For verification of deletion, the *PDC* gene was PCR-amplified using primers PDC.check.F and PDC.check.R from genomic DNA, and the PCR product was digested with *Eco*RI.

Successful deletion of *PDC* resulted in 2 bands on the agarose gel. Plasmid pVT36b-PDC was then cured using SC-FOA, resulting in strain SA/MAE1/pdcΔ/ura3Δ.

For *GPD* deletion, plasmid pVT36b-GPD was transformed into strain SA/MAE1/pdcΔ/ura3Δ. For verification of deletion, the *GPD* gene was PCR-amplified using primers GPD.check.F and GPD.check.R from genomic DNA, and the PCR product was digested with *Eco*RI. Successful deletion of *GPD* resulted in 2 bands on the agarose gel. Plasmid pVT36b-GPD was then cured using SC-FOA, resulting in strain SA/MAE1/pdcΔ/gpdΔ/ura3Δ. Uracil prototrophy was restored by integration of URA3p-*URA3*-PDC1t cassette, amplified using primers URA3.F and URA3.R from p101a, to strain SA/MAE1/pdcΔ/gpdΔ/ura3Δ, leading to strain SA/MAE1/pdcΔ/gpdΔ.

Strains SA/MAE1/pdcΔ/gpdΔ/X. The cassette amplified from p101a-PYC, p101a-MDH, p101a-FUMR, or p101a-FRD using primers URA3.F and URA3.R was transformed to strain SA/MAE1/pdcΔ/gpdΔ/ura3Δ. To express one more copy of the full reductive TCA pathway, the reductive TCA pathway cassette was isolated from p415-E using *Mlu*I and transformed to strain SA/MAE1/pdcΔ/gpdΔ/ura3Δ.

*Strain g3473*Δ. Plasmid pVT36b-g3473 was transformed into strain SA/MAE1/pdcΔ/gpdΔ/ura3Δ. For verification of deletion, g3473 was PCR-amplified from genomic DNA using primers g3473.check.F and g3473.check.R, and the PCR product was digested with *Xho*I. Successful deletion of g3473 resulted in 2 bands on the agarose gel. Plasmid pVT36b-GPD was then cured using SC-FOA, resulting in strain g3473Δ/ura3Δ. Uracil prototrophy was restored by integration of URA3p-*URA3*-PDC1t cassette, amplified using primers URA3.F and URA3.R from p101a, to strain g3473Δ/ura3Δ, leading to strain g3473Δ.

*Strain g3473Δ/g3068*Δ. Plasmid pVT36b-g3068 was transformed into strain g3473Δ. For verification of deletion, g3068 was PCR-amplified from genomic DNA using primers g3068.check.F and g3068.check.R, and the PCR product was digested with *Xho*I. Successful deletion of g3068 resulted in 2 bands on the agarose gel. Plasmid pVT36b-g3068 was then cured using SC-FOA, resulting in strain g3473Δ/g3068Δ/ura3Δ. Uracil prototrophy was restored by integration of URA3p-*URA3*-PDC1t cassette, amplified using primers URA3.F and URA3.R from p101a, to strain g3473Δ/g3068Δ/ura3Δ, leading to strain g3473Δ/g3068Δ.

*Strain g3473Δ/nde*Δ. Plasmid pVT36b-NDE was transformed into strain g3473Δ. For verification of deletion, *NDE* was PCR-amplified from genomic DNA using primers NDE.check.F and NDE.check.R, and the PCR product was digested with *Xho*I. Successful deletion of *NDE* resulted in 2 bands on the agarose gel. Plasmid pVT36b-NDE was then cured using SC-FOA, resulting in strain g3473Δ/ndeΔ/ura3Δ. Uracil prototrophy was restored by integration of URA3p-*URA3*-PDC1t cassette, amplified using primers URA3.F and URA3.R from p101a, to strain g3473Δ/ndeΔ/ura3Δ, leading to strain g3473Δ/ndeΔ.

Strain g3473Δ/PaGDH-DAK. The cassette TDH3p-*DAK*-g3376t-g853p-*PaGDH*-g3767t was PCR amplified using primers glycerol.int3.F and glycerol.int3.R and co-transformed with pVT36b-int3 into strain g3473Δ. Yeast colonies were screened for integration of TDH3p-*DAK*-g3376t-g853p-*PaGDH*-g3767t cassette by PCR using primers glycerol.check.F and int3.down.R. Plasmid pVT36b-int3 was then cured using SC-FOA, leading to strain g3473Δ/PaGDH-DAK/ura3Δ. Uracil prototrophy was restored by integration of URA3p-*URA3*-PDC1t cassette, amplified using primers URA3.F and URA3.R from p101a, to strain g3473Δ/PaGDH-DAK/ura3Δ, leading to strain g3473Δ/PaGDH-DAK.

Strain g3473Δ/ndeΔ/PaGDH-DAK. The cassette TDH3p-*DAK*-g3376t-g853p-*PaGDH*-g3767t was PCR amplified using primers glycerol.int3.F and glycerol.int3.R and co-transformed with pVT36b-int3 into strain g3473Δ/ndeΔ. Yeast colonies were screened for integration of TDH3p-*DAK*-g3376t-g853p-*PaGDH*-g3767t cassette by PCR using primers glycerol.check.F and int3.down.R. Plasmid pVT36b-int3 was then cured using SC-FOA, leading to strain g3473Δ/ndeΔ/PaGDH-DAK/ura3Δ. Uracil prototrophy was restored by integration of URA3p-*URA3*-PDC1t cassette, amplified using primers URA3.F and URA3.R from p101a, to strain g3473Δ/ndeΔ/PaGDH-DAK/ura3Δ, leading to strain g3473Δ/ndeΔ/PaGDH-DAK.

Strains g3473Δ/PaGDH-DAK/g3837Δ, g3473Δ/PaGDH-DAK/g1398Δ, and *g3473Δ/PaGDH-DAK/g2945Δ*. Plasmid pVT36b-g3837 was transformed into strain g3473Δ/PaGDH-DAK. For verification of deletion, g3837 was PCR-amplified from genomic DNA using primers g3837.check.F and g3837.check.R, and the PCR product was digested with *Xho*I. Successful deletion of g3837 resulted in 2 bands on the agarose gel. Plasmid pVT36b-g3837 was then cured using SC-FOA, resulting in strain g3473Δ/PaGDH-DAK/g3837Δ/ura3Δ. Uracil prototrophy was restored by integration of URA3p-*URA3*-PDC1t cassette, amplified using primers URA3.F and URA3.R from p101a, to strain g3473Δ/PaGDH-DAK/g3837Δ/ura3Δ, leading to strain g3473Δ/PaGDH-DAK/g3837Δ/ura3Δ. Strains g3473Δ/PaGDH-DAK/g1398Δ and g3473Δ/PaGDH-DAK/g2945Δ were constructed similarly.

Strain g3473Δ/PaGDH-DAK/ScSUC2. The cassette TDH3p-*ScSUC2*-g3376t was PCR amplified using primers p101a.int5.F and p101a.int5.R and co-transformed with pVT36b-int5 into strain g3473Δ/PaGDH-DAK/ura3Δ. Yeast colonies were screened for integration of TDH3p-*ScSUC2*-g3376t cassette by PCR using primers SUC2.check.F and int5.down.R. Plasmid pVT36b-int5 was then cured using SC-FOA, leading to strain g3473Δ/PaGDH-DAK/ScSUC2/ura3Δ. Uracil prototrophy was restored by integration of URA3p-*URA3*-PDC1t cassette, amplified using primers URA3.F and URA3.R from p101a, to strain g3473Δ/PaGDH-DAK/ScSUC2/ura3Δ, leading to strain g3473Δ/PaGDH-DAK/ScSUC2.

Supplementary Method 3. Piloting

# Lab scale calculations:

Impeller Reynolds Number (Re_i):

$$Re_i = \frac{\rho \times N \times D_i^2}{\mu} \quad \textbf{(1)}$$

ρ = density of fluid (assumed as 1000 kg.m^{-3}); N = Agitation rate in RPM; D_i = Impeller Diameter in m; and μ = fluid viscosity (taken as 1.793 × 10^{-3} Kg.m^{-1}.s^{-1})

Hence, for lab scale: $Re_i = \frac{1000(kg.m^{-3})\times(\frac{800}{60}s^{-1})\times(0.027)^2}{0.001793} = \frac{9.72}{0.001793}$ = 5421.081

Now, using the chart for disc type[3], the Power Number N_P = 5.

Thus, $P = N_P \times \rho \times N^3 \times D_i^5$ **(2)**

P = power input; N_P = Power number =5; ρ = density of fluid (assumed as 1000 kg.m^{-3}); N = Agitation rate in RPM; D_i = Impeller Diameter in m

Putting the values in equation 2, we get:

$P = 5 \times 1000(kg.m^{-3}) \times (800/60)^3 \times (0.027)^5$ = 5000×2370.37×1.43×10^{-8}

or P = 0.170061 W = (0.170061/745.7) hp = 2.28e^{-4} hp

Now, Aeration rate = 2 vvm = 2×2.5×10^{-4} m^3.min^{-1} = 5 ×10^{-4} m^3.min^{-1} = 8.33×10^{-6} m^3.sec^{-1}

$$\frac{P_{gassed}}{P_{ungassed}} = 0.10 \times (\frac{Q}{N.V_L})^{-0.25} \times (\frac{N^2 D_i^4}{g.B.V_L^{2/3}})^{-0.20} \quad \textbf{(3)}$$

B = impeller blade width = 0.0041 m

So, $(\frac{Q}{N.V_L})^{-0.25} = (\frac{8.33\times10^{-6}}{13.33\times2.5\times10^{-4}})^{-0.25}$ = 4.472

$(\frac{N^2 D_i^4}{g.B.V_L^{2/3}})^{-0.20} = (\frac{13.33^2\times0.027^4}{9.81\times0.0041\times(2.5\times10^{-4})^{2/3}})^{-0.20}$ = 1.1106

Thus, $\frac{P_{gassed}}{P_{ungassed}} = 0.10 \times 4.472 \times 1.1106$ = 0.4966

So, P_{gassed} = 0.4966×0.170061 W = 0.084 W = 0.0001126 hp

Also, $\frac{P_{gassed}}{V_L} = \frac{0.084}{2.5\times10^{-4}}$ = 338

(1) Scale-up criteria of same Re_i: Here, 1 and 2 represent lab and pilot scale, respectively.

$$\frac{\rho\times N_1\times D_1^2}{\mu} = \frac{\rho\times N_2\times D_2^2}{\mu} \quad \textbf{(4)}$$

$$N_1 \times D_1^2 = N_2 \times D_2^2 \quad \textbf{(5)}$$

Or, $N_2 = \frac{N_1\times D_1^2}{D_2^2} = \frac{13.33\times0.027^2}{0.1651^2}$ = 0.356 RPS = 21 RPM

This is very low as per reactor volume.

(2) On considering, P/V_L as scale-up parameter:

$$P_2 = P_1\frac{V_2}{V_1} \quad \textbf{(6)}$$

The volume ratio of the two geometrically similar vessels given as,

$$H_2 = H_1\frac{D_2}{D_1} \quad \textbf{(7)}$$

Here, D_1 and D_2 are Reactor diameter at two scales.

Hence, from 6 and 7 we have, $\frac{V_2}{V_1} = (\frac{D_2}{D_1})^3$ **(8)**

So, the increase in power required on the large scale in terms of the impeller diameter ratio is

$$P_2 = P_1(\frac{D_2}{D_1})^3 \quad \textbf{(9)}$$

A constant power number implies that P is proportional to N^3D^5. Since under constant oxygen transfer conditions, the ratio of cube of the impeller diameters equals the ratio of the power input.

from, $\frac{P_2}{P_1} = \frac{N_2^3D_2^5}{N_1^3D_1^5} = \frac{D_2^3}{D_1^3}$ we get, $\frac{N_1}{N_2} = (\frac{D_2}{D_1})^{2/3}$ **(10)**

$$\frac{13.33}{N_2} = (\frac{0.1651}{0.027})^{2/3} \quad \textbf{(11)}$$

$\mathbf{N}_2$ = 13.33/3.344 = 3.986 RPS = 239.17 RPM

Hence, from scale-up criteria 1 and 2, we suggested RPM in the range of 100 < N_2 < 300 (or x g in the range of 1 to 8) and vary aeration to maintain DO set point 8%.

Supplementary Method 4. Optimization of crystallization operational parameters

The operational parameters that can influence the SA crystallization from fermentation broth were identified as (i) seed loading, (ii) seed loading temperature, (iii) time, and (iv) agitation. The crystallization temperature was fixed at 0 °C. Also, to investigate the effect of initial SA concentration on the SA recovery, a synthetic solution of 100 g/L, 200 g/L and 250 g/L SA concentration (pH 3) was prepared. The range of these crystallization operational parameters employed to optimize SA recovery was presented in Supplementary Table 6.

First, the crystallization operational parameters were optimized for a synthetic solution having an SA concentration of 100 g/L. Then these optimized parameters were applied to investigate the SA recovery from a higher titer value of 200 g/L and 250 g/L. The obtained result described below in brief:

To study the impact of seed loading and seed loading temperature on SA recovery, a synthetic solution of SA concentration 100 g/L at pH 3 was utilized. The pH was maintained by adding 1 M H_2SO_4 solution. The effect of seed loading temperature is investigated in the range of 10 to 20 °C at 200 RPM in a temperature-controlled shaker. The seed loading and processing time of crystallization were fixed at 1% w/w and 2 h, respectively. At 1% seed loading and seeding temperature of 10 °C, the SA recovery of 31.76% (±0.3) was observed. Also, at seed loading temperatures of 15 °C and 20 °C, the obtained value of SA recovery was 25.17 (±0.16) and 24.27 (±0.11), respectively. This indicates that the initiation of seed load at higher temperatures (>10 °C) results in lower SA recovery. Thus, for further studies, the seed loading temperature was fixed at 10 °C. Then, the influence of % seed loading was studied by varying in the range of 0.5 to 2 % w/v at 200 RPM for 2h of process time. The obtained result shows SA recovery of 21.08 (±0.18), 31.76 (±0.3), and 32.57 (±0.34) % at 0.5%, 1%, and 2% seed loading, respectively. Thus, only a marginal increment in SA recovery was observed on increasing a seed loading from 1% to 2%. Hence, a seed loading of 1% and seed loading temperature of 10 °C was fixed for investigating the effect of process time at 200 RPM.

The synthetic solution of SA concentration 10 g/100 mL at pH 3 is subjected to crystallization for 2h, 4 h, and 6h. The SA recovery of 31.76 (±0.3), 38.81 (±0.21), and 41.38 (±0.15) is obtained for 2h, 4 h, and 6 h of process time. Thus, from increasing the processing time 4 h to 6 h, only a marginal increment of ~3% in SA recovery was observed. Therefore, a processing time of 4 h was fixed to investigate the impact of RPM on SA recovery. The agitation is varied in the range of 50 to 200 RPM while %seed loading, seed loading temperature, and crystallization time are maintained at 1%, 10 °C, and 4 h, respectively. The obtained values of SA % recovery was 24.27(±0.12), 25.17(±0.41), and 31.76 (±0.3) at 50, 100, and 200 RPM, respectively. Thus, as the maximum SA % recovery was obtained at 200 RPM hence, for further studies, an agitation rate of 200 RPM (or 0.56 x g) was selected.

Thus, for a synthetic solution consisting of 100 g/L of SA, the optimized values of seed loading, seed loading temperature, time, and agitation were obtained as 1% w/v, 10 °C, 4 hours, and 200 RPM (or 0.56 x g). Now the influence of the initial SA titer value on SA % recovery was studied by utilizing the synthetic solutions of SA concentrations 200 g/L and 250 g/L, respectively. The pH is maintained at 3 and the optimized parameters of 1% w/v, 10 °C, 4 hours, and 200 RPM is implemented. The obtained result indicates a % SA recovery of 73.86 (±0.09) and 90.19% (±0.24) for initial SA concentrations of 200 g/L and 250 g/L, respectively. In other studies, a similar SA % recovery of 72.4% and 74.8% is obtained using a simulated solution consisting of 110 g/L and 150 g/L SA through a direct crystallization approach. This indicates higher titer value of SA results in a higher % recovery of SA via direct crystallization approach.

Supplementary Table 1. Distributions of results from the uncertainty analyses for the *pilot batch scenario* in this work for MPSP, GWP_{100}, and FEC at alternative numbers of Monte Carlo simulations (1000, 2000, 3000, and 5000). Source data are provided as a Source Data file.

**Percentile**	**MPSP ($/kg)**				**GWP_{100} (kg CO_2-eq./kg)**				**FEC (MJ/kg)**			
	1000	2000	3000	5000	1000	2000	3000	5000	1000	2000	3000	5000
5th	1.23	1.23	123	1.23	1.23	1.22	1.22	1.23	-7.02	-7.08	-7.17	-6.90
10th	1.26	1.26	1.26	1.26	1.35	1.32	1.32	1.33	-4.40	-5.54	-5.40	-5.41
25th	1.31	1.31	1.31	1.31	1.50	1.50	1.51	1.50	-2.56	-2.75	-2.64	-2.77
50th	1.37	1.37	1.37	1.37	1.70	1.71	1.70	1.70	0.145	0.265	0.207	0.140
75th	1.44	1.44	1.44	1.44	1.89	1.91	1.90	1.90	2.68	2.93	2.83	2.94
90th	1.50	1.50	1.50	1.50	2.06	2.07	2.08	2.07	5.03	5.26	5.21	5.15
95th	1.53	1.54	1.54	1.54	2.17	2.17	2.20	2.18	6.41	6.47	6.75	6.50

Supplementary Table 2. Spearman's rank order correlation coefficients with respect to minimum product selling price (MPSP), 100-year global warming potential (GWP100), and fossil energy consumption (FEC) for the *pilot batch scenario*.

Parameter name	Spearman's rank order correlation coefficient with respect to …		
	MPSP	GWP_{100}	FEC
Gypsum unit price	0.02	0.03	0.02
Plant uptime	-0.38	0.01	0.02
Feedstock unit price	0.39	-0.01	-0.01
Natural gas unit price	0.00	-0.03	-0.04
Electricity unit price	-0.02	-0.04	-0.04
Mono-ethanolamine unit price	-0.01	0.02	0.02
Liquid CO2 unit price	0.02	-0.05	-0.06
Lime unit price	0.05	-0.02	-0.02
Diammonium sulfate unit price	0.04	0.02	0.00
Magnesium sulfate unit price	0.01	0.06	0.05
Feedstock capacity	-0.31	0.01	0.03
CSL loading	0.02	0.25	0.07
Seed train fermentation ratio	-0.01	0.00	-0.01
Inoculum ratio	0.06	0.03	0.00
Succinic acid yield	-0.60	0.32	0.63
Succinic acid titer	-0.30	-0.62	-0.49
Succinic acid productivity	-0.16	-0.02	0.00
Issatchenkia orientalis yield	-0.07	-0.12	-0.11
Base required: succinic acid produced	0.06	0.04	-0.02
Diammonium sulfate required: succinic acid produced ratio	0.05	0.08	0.06
Magnesium sulfate required: succinic acid produced ratio	0.02	-0.03	-0.03
Air required: succinic acid produced ratio	0.11	0.02	0.01
CO2 required: succinic acid produced ratio	0.06	0.07	0.05
Fermentation power per unit volume of reactor	-0.01	0.07	0.08
Crystallization output concentration multiplier	0.09	0.05	0.02
Crystallization pressure filter recovery	-0.08	-0.01	0.01
Product succinic storage time	0.03	0.03	0.01
Boiler efficiency	-0.15	-0.63	-0.56

Supplementary Table 3. **Reported fermentation performance assumptions and minimum product selling prices (MPSPs) reported in the literature for techno-economic analysis of bio-based succinic acid production and used for comparison in this study.**

Fermentation titer (g/L)	Fermentation productivity (g/L/h)	Fermentation yield (g/g-sugars)	Reported MPSP ($/kg)	MPSP adjusted to 2016$ and IRR of 10% ($/kg)	Reference
31.7	0.67	0.68	1.13	1.08	7
37.2	0.79	0.79	4.42	3.63	8
66.7	0.92	0.60	3.61	2.95	9
56.4	1.08	0.73	1.60	1.51	10
55.8	0.77	0.96	1.35	1.3	11
41.46	0.84	0.82	2.70	2.74	12
21.81	0.3	0.45	2.32	2.05	13
13.7	1.108455	1.10	2.26	2.49	14
63.5	2.54	1.05	1.23	1.27	15

Supplementary Table 4. Reported 100-year global warming potential (GWP_{100}) and fossil energy consumption (FEC) values for fossil-based and bio-based succinic acid used for comparison in this study.

Feedstock	Reported GWP100 (kg CO_2-eq./kg)	GWP100 after adding end-of-life emissions of 1.49 kg CO_2-eq./kg (if not originally included) (kg CO_2-eq./kg)	Reported FEC (MJ/kg)	Reference
Fossil fuels	12.13	12.13	112	10
	1.78	3.27	60.8	17
	1.94	3.43	59.2	
	8.82	10.31	124.3	
Corn stover	1.83	1.83	27.7	10
	3.25	3.25	42.1	
	0.704	2.20	26.0	18
Corn	0.88	2.37	32.7	17

Supplementary Table 5. Strains used in this study.

Strains	Features	Sources
E. coli DH5α	Cloning host	NEB
S. cerevisiae HZ848	Host used for DNA assembly	Ref. 1
SD108/ura3Δ	*I. orientalis* SD108 with uracil autotrophy	Ref. 5
SA	Io−ura3/*ura3*−::ura3-succinic acid biosynthetic pathway	Ref. 5
SA/MAE1	SD108 with overexpression of reductive TCA pathway and *SpMAE1*	This study
SA/MAE1/pdcΔ/gpdΔ	SA/MAE1with *GPD* and *PDC* deletions	This study
SA/MAE1/pdcΔ/gpdΔ/X	SA/MAE1/pdcΔ/gpdΔ with overexpression of another copy of PYC, MDH, FUMR, FRD, or full reductive TCA pathway	This study
g3473Δ	SA/MAE1/pdcΔ/gpdΔ with g3473 deletion	This study
g3473Δ/g3068Δ	g3473Δ with g3068 deletion	This study
g3473Δ/ndeΔ	g3473Δ with *NDE* deletion	This study
g3473Δ/PaGDH-DAK	g3473Δ with overexpression of *PaGDH* and *DAK*	This study
g3473Δ/ndeΔ/PaGDH-DAK	g3473Δ/ndeΔ with overexpression of *PaGDH* and *DAK*	This study
g3473Δ/PaGDH-DAK/g3837Δ	g3473Δ/PaGDH-DAK with g3837 deletion	This study
g3473Δ/PaGDH-DAK/g1398Δ	g3473Δ/PaGDH-DAK with g1398 deletion	This study
g3473Δ/PaGDH-DAK/g2945Δ	g3473Δ/PaGDH-DAK with g2945 deletion	This study
g3473Δ/PaGDH-DAK/ScSUC2	g3473Δ/PaGDH-DAK with overexpression of ScSUC2	This study

Supplementary Table 6. Plasmids used in this study.

Plasmids	Features	Sources
pRS416	*S. cerevisiae* plasmid containing *URA3* marker and ARS/CEN	NEB
pVT36b	*I. orientalis* plasmid for gene deletion	Ref. 6
pRS415-E	pRS415 with *URA3*, *PYC*, *MDH*, *FUMR* and *FRD* expression cassettes added	Ref. 5
p101a	Helper plasmid to clone genes into the TDH3p-g3376t cassette; constructed by DNA assembler	This study
p102	Helper plasmid to clone genes into the g853p-g3767t cassette; constructed by DNA assembler	This study
pRS416-SA-site1	pRS416 harboring reductive TCA pathway flanked with homology arms for integration to site 1; constructed by DNA assembler	This study
p102-MAE1	p102 with *SpMAE1* insertion	This study
p101a-DAK	p101a with *DAK* insertion	This study
p101a-PYC	p101a with *PYC* insertion	This study
p101a-MDH	p101a with *MDH* insertion	This study
p101a-FUMR	p101a with *FUMR* insertion	This study
p101a-FRD	p101a with *FRD* insertion	This study
p102-PaGDH	p102 with *PaGDH* insertion	This study
p416-PaGDH-DAK	pRS416 harboring TDH3p-*DAK*-g3376t and g853p-*PaGDH*-g3767t cassettes	This study
p101a-SUC2	p101a with *ScSUC2* insertion	This study
pVT36b-PDC	pVT36b with insertion of gBlock for *PDC* deletion	This study
pVT36b-GPD	pVT36b with insertion of gBlock for *GPD* deletion	This study
pVT36b-g3473	pVT36b with insertion of gBlock for g3473 deletion	This study
pVT36b-g3068	pVT36b with insertion of gBlock for g3068 deletion	This study
pVT36b-NDE	pVT36b with insertion of gBlock for *NDE* deletion	This study
pVT36b-g3837	pVT36b with insertion of gBlock for g3837 deletion	This study
pVT36b-g1398	pVT36b with insertion of gBlock for g1398 deletion	This study
pVT36b-g2945	pVT36b with insertion of gBlock for g2945 deletion	This study
pVT36b-int1	pVT36b with insertion of spacer for integration at site 1	This study
pVT36b-int2	pVT36b with insertion of spacer for integration at site 2	This study
pVT36b-int3	pVT36b with insertion of spacer for integration at site 3	This study
pVT36b-int5	pVT36b with insertion of spacer for integration at site 5	This study

Supplementary Table 7. gBlocks used in this study.

gBlocks	Sequences (5' to 3')
PDC	CTTTGGTCTCCTGCATCAGCATAAGAACCTGCAATGGCATTGACGGCAGACAATTCACCGA CACCGAATTCTGATTAGGGATGCAAATCCATTGATTCTTGCATAACCATCAGCTTCGTAGC AATTCACCGACACCAAAGGGTTTGGAGACCTTTC
GPD	CTTTGGTCTCCTGCAAAGACTATCCGGAACATCCGTTCAAGGTGACGGTTGTTGGTTCCGG TAACGAATTCGTACAATTGCCAAGGTTATAGCGGAAAACACCGTTGAGAGACCTCGTCAAT TGTTGGTTCCGGTAACTGGGTTTGGAGACCTTTC
g3473	CTTTGGTCTCCTGCACAAGATTTACTGAGTTGTTCCCAACAAAGCAGTCGATGGCTGCCAA CAAGCTCGAGTTTAAGAATGATTGGTTTCAAACAATGGCTCTTGATTTTATCTGGGTTCCC ACCTTTTGAATCCTCTTCCGTTTGGAGACCTTTC
g3068	CTTTGGTCTCCTGCACCAGATTCACTGAGCTATTGCCAACAAAGCAACAGATGAAACAAAA TAAACTCGAGCTTGAGAATGATGGGTCGTAGACAATGGCTGATGTTTCTGAGTGCCTTCTC ATTTGTTGAATCCATTGCCGTTTGGAGACCTTTC
NDE	CTTTGGTCTCCTGCAGTAAATACCTTTGGCATTCCTGGTATTCCTGAATATGCTTCTTACC TTAACTCGAGTAAGACAAAAGTTATTCAACCAAATTGAAGCTTCTAGGTTGTTGCCAAAGA GAAGCAAATGATGCTACCGGTTTGGAGACCTTTC
g3837	CTTTGGTCTCCTGCATGATTCCAACATACGTTACGCAGATCCCAACGGGTAAGGAGAAGGG TGTCCTCGAGACACTTTTGAGTTGAAGCAGTCCAAGAACCCGATTCCCGTGGACTTGATGC TTTTAGCAGCCGATTTGGGGTTTGGAGACCTTTC
g1398	CTTTGGTCTCCTGCACAACTGAAAAGTTGGAAGAAATCACAAACCATTTTGTTGAAGAACT CGAACTCGAGTAATATTCCAATGAATCCTACTTGGGTTATGGATTATCCATCAGGTTCCGA AGGGTATTTCTCCAGCCGGGTTTGGAGACCTTTC
g2945	CTTTGGTCTCCTGCATGAATGTTGCTTGGGTCATGGAATACCCAACAGGTGACGAAACGGG CGACCTCGAGTACCAATTTGAGGGTCGTTATTGTCCATTTGAAGGGTAAAGGTCAGATGGT ACCTAGCTCTGGATATGGGGTTTGGAGACCTTTC

Supplementary Table 8. Codon-optimized genes used in this study.

Genes	Sequences (5’ to 3’)
SpMAE1	ATGGGCGAGTTGAAAGAGATTTTAAAGCAAAGATACCACGAATTATTAGATTGGAACGTTA<br>AGGCTCCACACGTTCCATTGTCCCAGAGATTGAAACACTTCACTTGGTCCTGGTTCGCTTG<br>CACAATGGCGACCGGCGGAGTGGGACTTATCATCGGATCCTTTCCATTCAGATTCTACGGC<br>TTGAACACGATCGGAAAGATCGTCTACATCTTACAGATTTTCTTATTCTCCCTTTTCGGTA<br>GTTGTATGTTGTTCAGATTCATCAAGTACCCATCGACAATTAAAGACTCTTGGAATCACCA<br>CCTCGAGAAATTATTTATCGCAACCTGCTTATTGTCGATTTCAACCTTTATTGATATGCTC<br>GCAATCTATGCTTACCCCGACACTGGTGAATGGATGGTTTGGGTTATCCGTATTTTATACT<br>ATATCTATGTCGCTGTTTCCTTCATCTACTGTGTCATGGCCTTCTTCACTATCTTTAACAA<br>TCACGTTTACACTATCGAGACAGCCTCGCCAGCATGGATCTTACCCATCTTTCCACCAATG<br>ATCTGCGGAGTTATCGCCGGTGCTGTTAACTCAACTCAGCCAGCGCACCAGCTTAAGAACA<br>TGGTGATTTTCGGAATTTTGTTCCAGGGTTTGGGATTCTGGGTGTACCTCTTGTTGTTCGC<br>AGTTAACGTTCTCAGATTCTTCACAGTCGGTTTGGCTAAGCCACAGGACAGACCAGGAATG<br>TTCATGTTCGTGGGACCCCCTGCCTTTAGTGGCTTAGCTTTGATCAACATCGCAAGAGGCG<br>CGATGGGTTCAAGACCCTACATCTTCGTCGGTGCAAATTCTTCGGAATACTTAGGCTTCGT<br>CTCCACTTTCATGGCAATCTTCATCTGGGGCTTGGCCGCATGGTGCTATTGCTTAGCTATG<br>GTCAGTTTCCTTGCTGGTTTCTTTACACGTGCGCCATTAAAATTCGCATGCGGTTGGTTCG<br>CCTTTATCTTTCCAAATGTTGGATTCGTGAACTGCACAATCGAAATTGGCAAGATGATCGA<br>CTCAAAGGCATTTCAGATGTTCGGTCACATTATCGGTGTTATCTTGTGCATCCAATGGATT<br>TTATTGATGTACTTGATGGTTAGAGCATTCCTTGTTAACGACTTGTGTTACCCAGGTAAGG<br>ATGAGGACGCGCACCCACCACCTAAGCCTAACACTGGAGTTTTGAATCCCACTTTTCCACC<br>AGAGAAGGCGCCAGCTTCGTTAGAGAAGGTTGACACGCACGTTACTTCCACAGGTGGCGAG<br>TCCGACCCACCATCATCAGAGCACGAGTCCGTCTAG
PaGDH	ATGAAAGGTTTACTTTACTATGGTACCAACGACATACGTTATAGTGAAACAGTACCTGAAC<br>CAGAGATTAAAAACCCTAATGATGTTAAGATCAAAGTTTCTTATTGTGGATATGTGGTAC<br>TGATTTAAAAGAATTCACTTACTCAGGGGGACCCGTCTTTTTTCCTAAACAGGGAACAAAA<br>GATAAGATTTCCGGGTATGAATTACCATTATGTCCCGGACATGAGTTTTCAGGTACTGTCG<br>TGGAAGTTGGTAGTGGTGTCACGTCTGTTAAGCCTGGAGACAGAGTTGCTGTTGAAGCAAC<br>GTCACATTGCTCAGATCGTTCTAGATACAAGGACACTGTTGCTCAAGATTTGGGATTATGT<br>ATGGCATGTCAGTCAGGATCACCAAACTGTTGTGCCAGTTTGTCTTTTTGTGGTCTAGGAG<br>GTGCCTCTGGTGGTTTCGCTGAATATGTAGTCTACGGTGAAGATCACATGGTGAAGTTACC<br>CGATTCAATCCCTGACGATATTGGTGCCTTGGTTGAACCGATCTCTGTTGCATGGCATGCA<br>GTAGAAAGGGCTAGATTTCAGCCCGGTCAAACAGCACTTGTGCTAGGCGGAGGTCCAATCG<br>GTCTTGCAACCATACTTGCTCTACAAGGTCATCATGCAGGTAAGATTGTGTGTTCAGAACC<br>AGCTCTAATCAGGAGACAATTTGCAAAAGAACTTGGTGCTGAGGTCTTTGACCCTAGTACC<br>TGTGATGACGCCAACGCTGTGTTGAAAGCAATGGTTCCGGAAAATGAGGGTTTTCATGCAG<br>CTTTCGATTGTTCTGGAGTACCACAGACCTTTACTACCTCTATTGTGGCTACGGGTCCATC<br>GGGAATTGCTGTCAATGTTGCAGTTTGGGGTGATCATCCAATCGGCTTTATGCCTATGAGT<br>CTAACCTATCAAGAAAAATATGCAACTGGCTCAATGTGCTATACCGTGAAAGATTTCCAGG<br>AAGTTGTCAAAGCATTAGAAGATGGGTTGATTTCTCTTGACAAGGCAAGGAAGATGATTAC<br>TGGCAAGGTTCATTTGAAGGATGGGGTCGAGAAGGGTTTTAAACAATTGATCGAACATAAA<br>GAGAACAATGTTAAGATCCTAGTGACGCCCAATGAAGTAAGTTAG

Supplementary Table 9. **Design specifications of the reactors utilize in the present study.**

Parameter	Lab scale	Pilot scale
Nominal volume (L)	0.35	75
Maximum working volume according to the manufacture (L)	0.25	60
Actual working volume (L)	e-2	30 and 60
Tank diameter (D_T, m)	0.09	0.34925
Tank height (H_T, m)	0.36	1.04775
Impeller type	2x Rushton-type	Rushton
Impeller diameter (D_I, m)	0.027	0.1651
Number of impellers	2	3
H_T/ D_T	4	3
D_I/ D_T	0.3	0.473
H_I/ D_T	1.333	Not calculated
Aeration rate (vvm)	2VVM air / 0.2VVM CO_2	Varies to maintain 8% DO
Tip speed (m/s)	2.262	0.8648 to 1.73
Stirrer speed (RPM)	800	100 to 200
Stirrer speed (x g)	10	1 to 4

Supplementary Table 10. The ranges of crystallization operational parameters.

Operational parameters	Range		
Seed loading (% of solute w/v)	0.5%	1%	2%
Seed loading temperature (°C)	10	15	20
Crystallization time (h)	2	4	6
Agitation (RPM)	50	100	200
Agitation (x *g*)	0.035	0.14	0.56

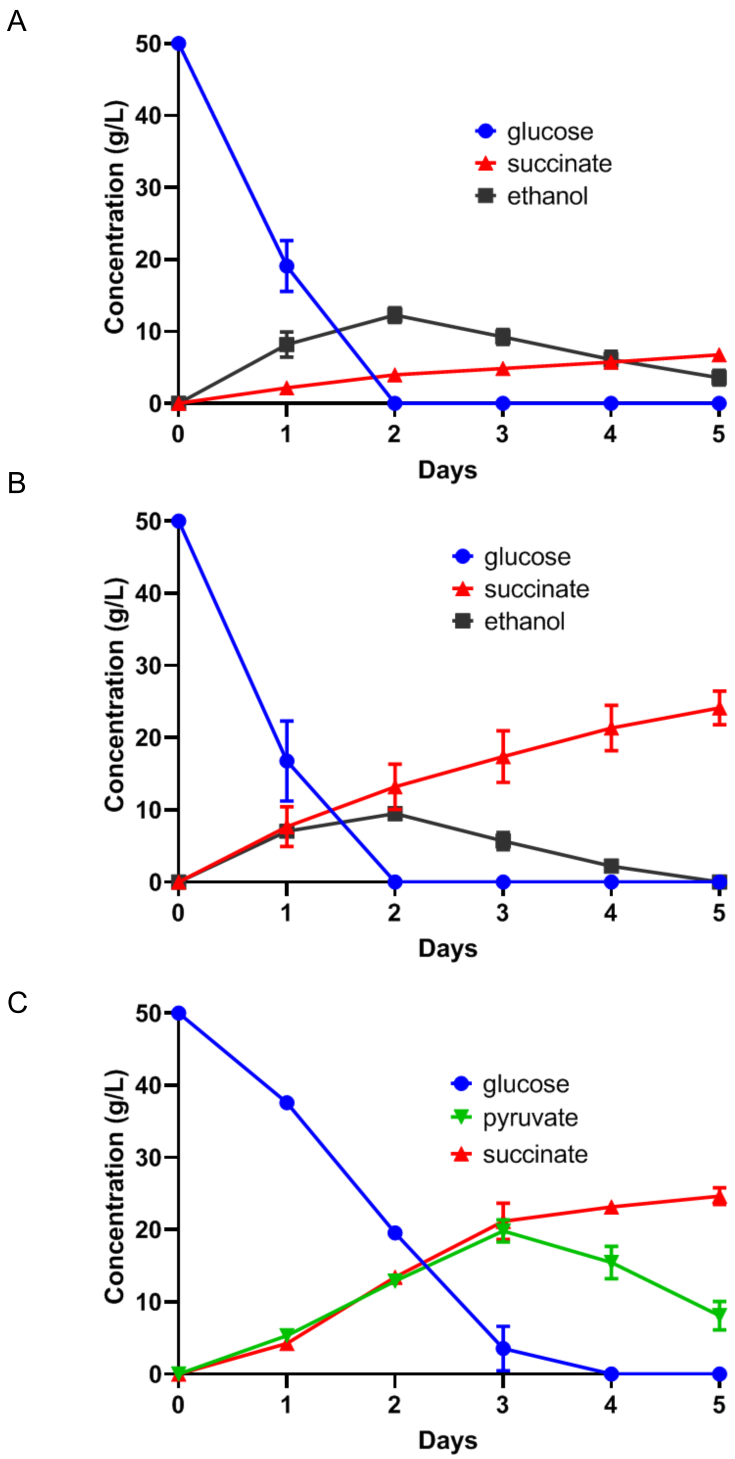

Supplementary Figure 1. Fermentation profiles of various strains in SC-URA medium with 50 g/L glucose under oxygen-limited condition in shake flasks. A. Fermentation of strain SA. Error bars, mean ± SD ($n = 3$ biologically independent samples). **B**. Fermentation of strain SA/MAE1. Error bars, mean ± SD ($n = 3$ biologically independent samples). **C**. Fermentation of strain SA/MAE1/pdcΔ/gpdΔ. Error bars, mean ± SD ($n = 3$ biologically independent samples). Source data are provided as a Source Data file.

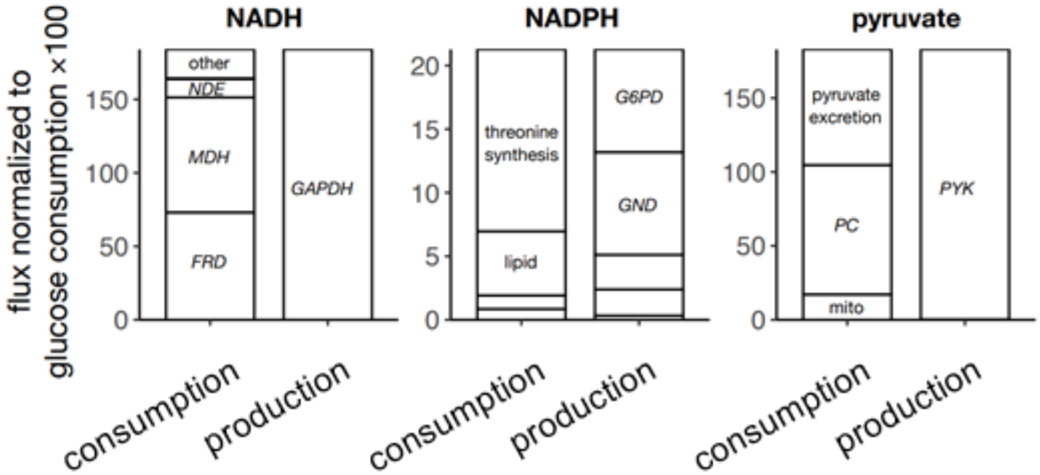

Supplementary Figure 2. Metabolic flux analysis of strain SA/MAE1/pdcΔ/gpdΔ. Source data are provided as a Source Data file.

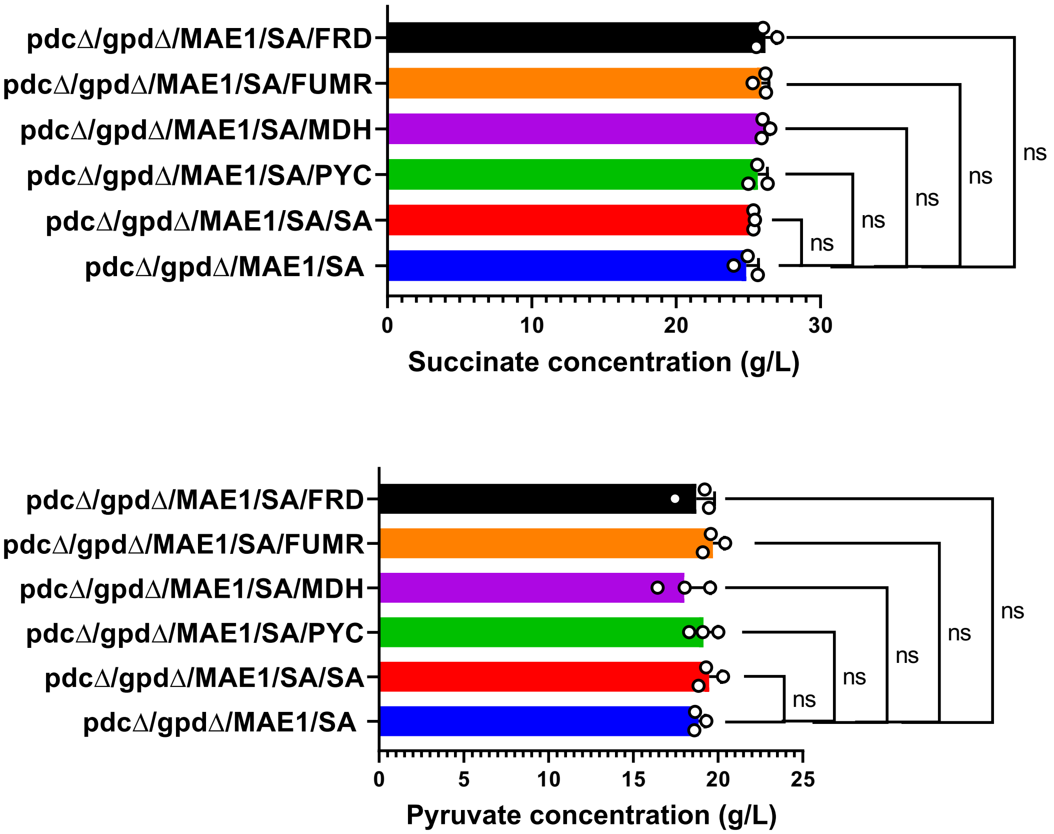

Supplementary Figure 3. Comparison of succinate and pyruvate titers between strain SA/MAE1/pdcΔ/gpdΔ and derived strains with one more copy of rTCA pathway or individual genes in the rTCA pathway. Error bars, mean ± SD ($n = 3$ biologically independent samples). ns = not significant, calculated by two-tailed unpaired t-test. Source data are provided as a Source Data file.

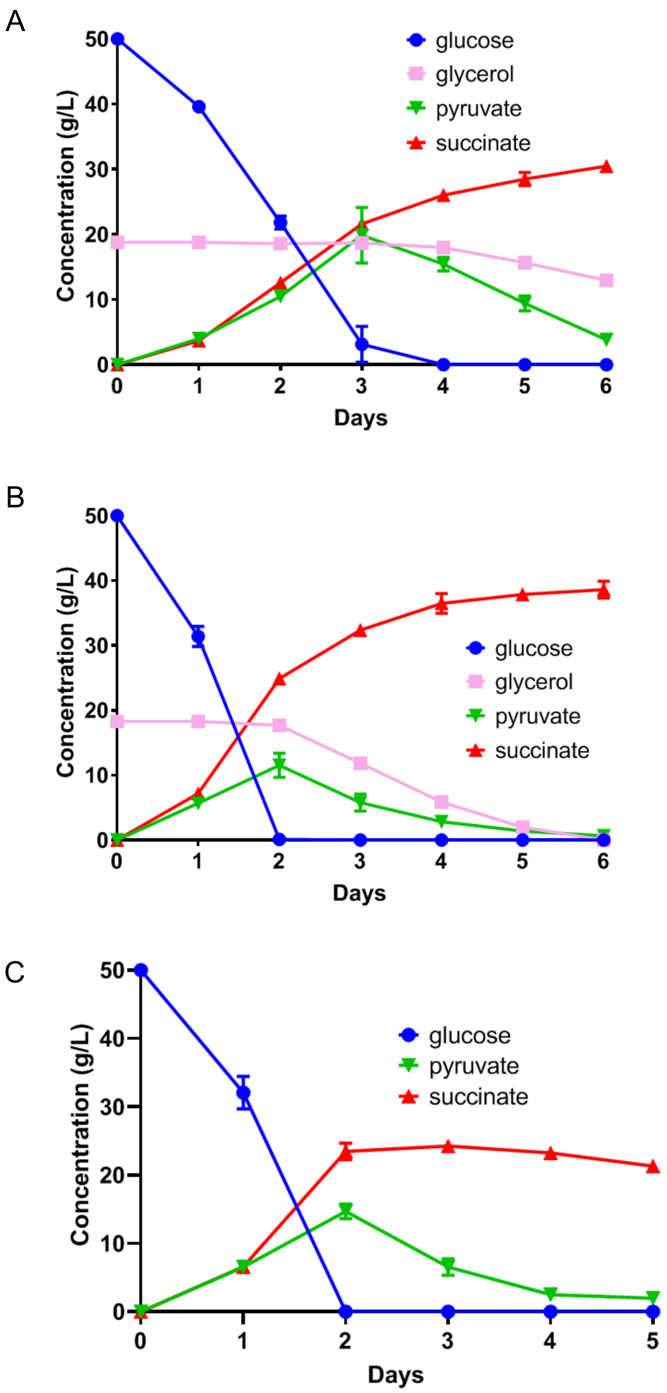

Supplementary Figure 4. Fermentation profiles of strain SA/MAE1/pdcΔ/gpdΔ. A. Fermentation profiles in SC-URA medium with 50 g/L glucose and 20 g/L glycerol in shake flasks under oxygen-limited condition. Error bars, mean ± SD (n = 3 biologically independent samples). **B**. Fermentation profiles in SC-URA medium with 50 g/L glucose and 20 g/L glycerol in shake flasks under aerobic condition. Error bars, mean ± SD (n = 3 biologically independent samples). **C**. Fermentation profiles in SC-URA medium with 50 g/L glucose in shake flasks under aerobic condition. Error bars, mean ± SD (n = 3 biologically independent samples). Source data are provided as a Source Data file.

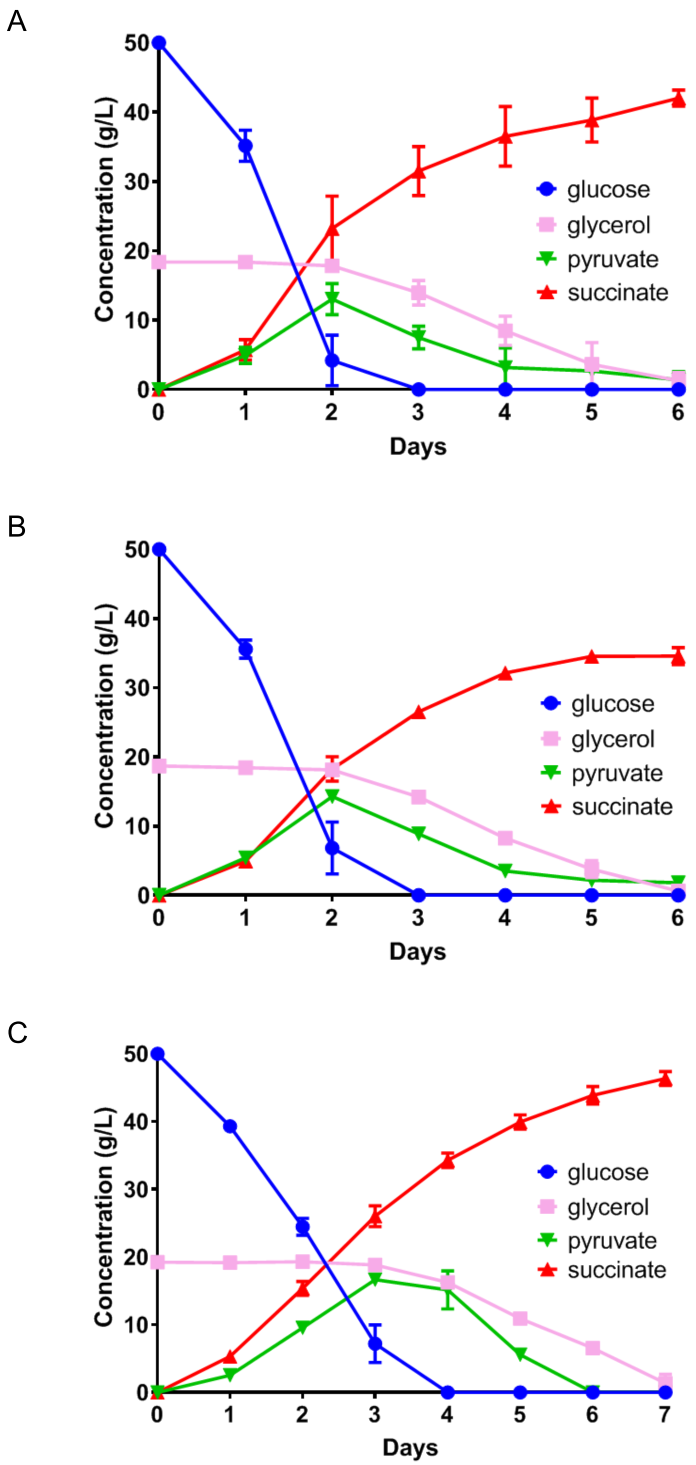

Supplementary Figure 5. Fermentation profiles of various strains in SC-URA medium with 50 g/L glucose and 20 g/L glycerol under aerobic condition in shake flasks. A. Fermentation profile of strain g3473Δ. Error bars, mean ± SD (*n* = 3 biologically independent samples). **B**. Fermentation profile of strain g3473Δ/g3068Δ. Error bars, mean ± SD (*n* = 3 biologically independent samples). **C**. Fermentation profile of strain g3473Δ/ndeΔ. Error bars, mean ± SD (*n* = 3 biologically independent samples). Source data are provided as a Source Data file.

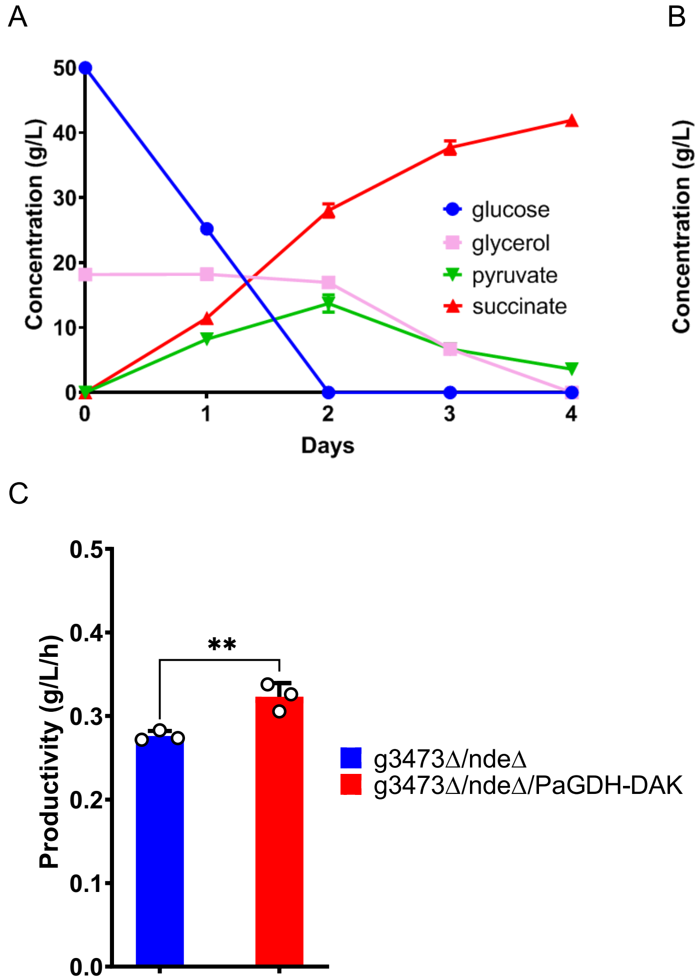

Supplementary Figure 6. Fermentation profiles of various strains in SC-URA medium with 50 g/L glucose and 20 g/L glycerol under aerobic condition in shake flasks. A. Fermentation profile of strain g3473Δ/PaGDH-DAK. Error bars, mean ± SD (n = 3 biologically independent samples). **B**. Fermentation profile of strain g3473Δ/ndeΔ/PaGDH-DAK. Error bars, mean ± SD (n = 3 biologically independent samples). **C**. Comparison of productivity between strain g3473Δ/ndeΔ and strain g3473Δ/ndeΔ/PaGDH-DAK. Error bars, mean ± SD (n = 3 biologically independent samples). **$P < 0.01$, calculated by two-tailed unpaired t-test. Source data are provided as a Source Data file.

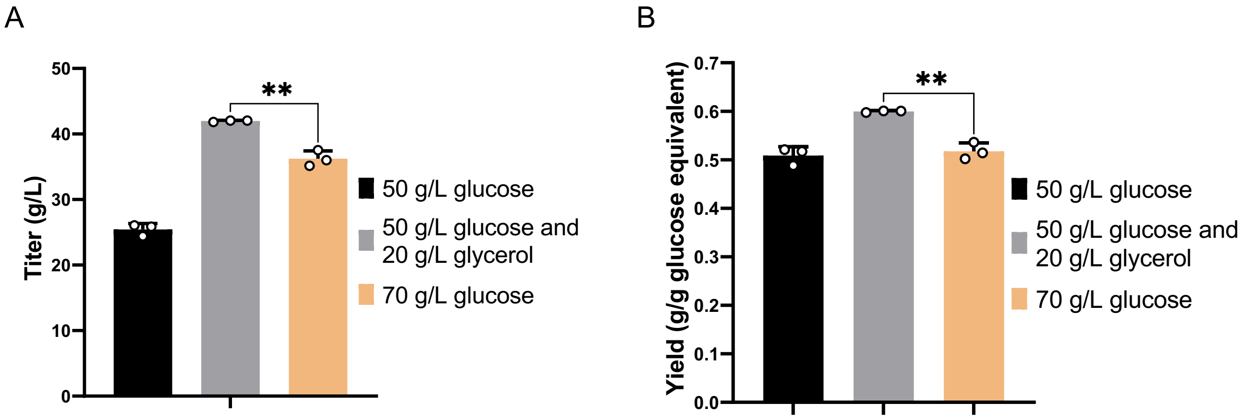

Supplementary Figure 7. Comparison between using only glucose and using both glucose and glycerol for production of SA. **A**. Titer. Error bars, mean ± SD ($n = 3$ biologically independent samples). $^{**}P < 0.01$, calculated by two-tailed unpaired t-test. **B**. Yield. Error bars, mean ± SD ($n = 3$ biologically independent samples). $^{**}P < 0.01$, calculated by two-tailed unpaired t-test. All fermentations were performed using strain g3473Δ/PaGDH-DAK in SC-URA medium with 50 g/L glucose, 50 g/L glucose and 20 g/L glycerol, and 70 g/L glucose under aerobic condition in shake flasks. Source data are provided as a Source Data file.

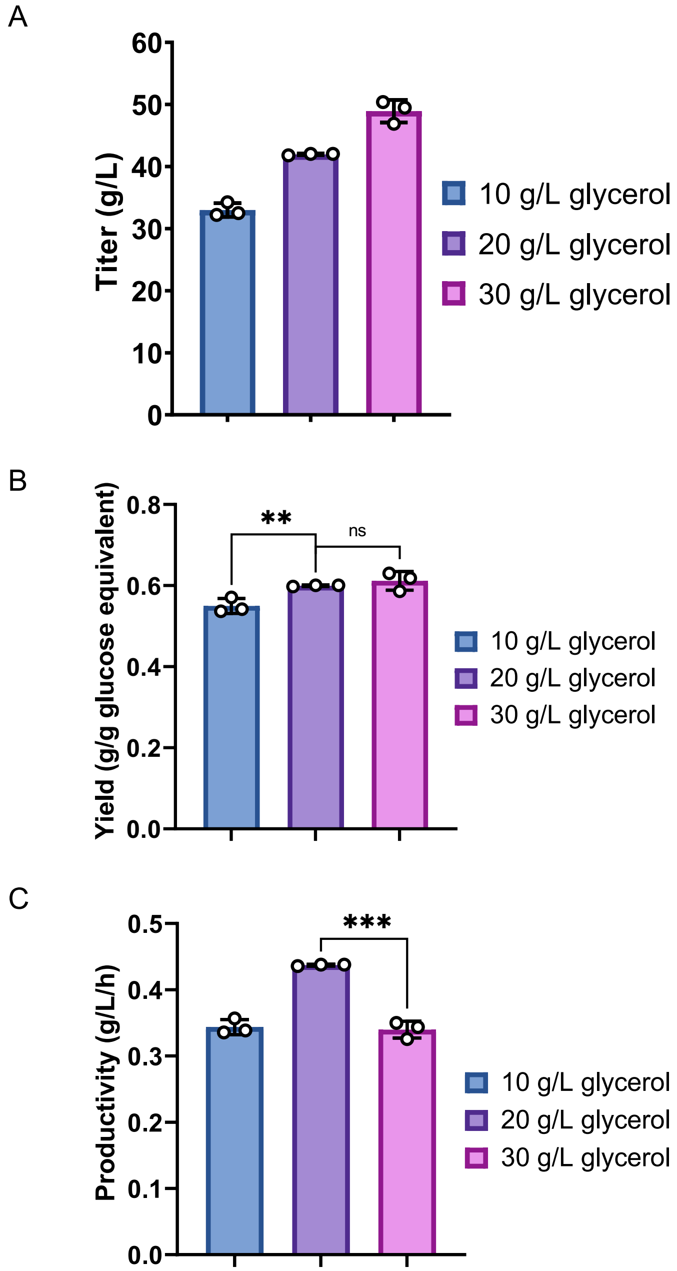

Supplementary Figure 8. Comparison of SA production in strain g3473Δ/PaGDH-DAK using 50 g/L of glucose and different glycerol concentrations of 10 g/L, 20 g/L, and 30 g/L. All fermentations were performed in SC-URA medium under aerobic condition in shake flasks. **A**. Titer. Error bars, mean ± SD ($n = 3$ biologically independent samples). **B**. Yield. Error bars, mean ± SD ($n = 3$ biologically independent samples). $^{**}P < 0.01$, ns = not significant, calculated by two-tailed unpaired *t*-test. **C**. Productivity. Error bars, mean ± SD ($n = 3$ biologically independent samples). $^{***}P < 0.001$, calculated by two-tailed unpaired *t*-test. Source data are provided as a Source Data file.

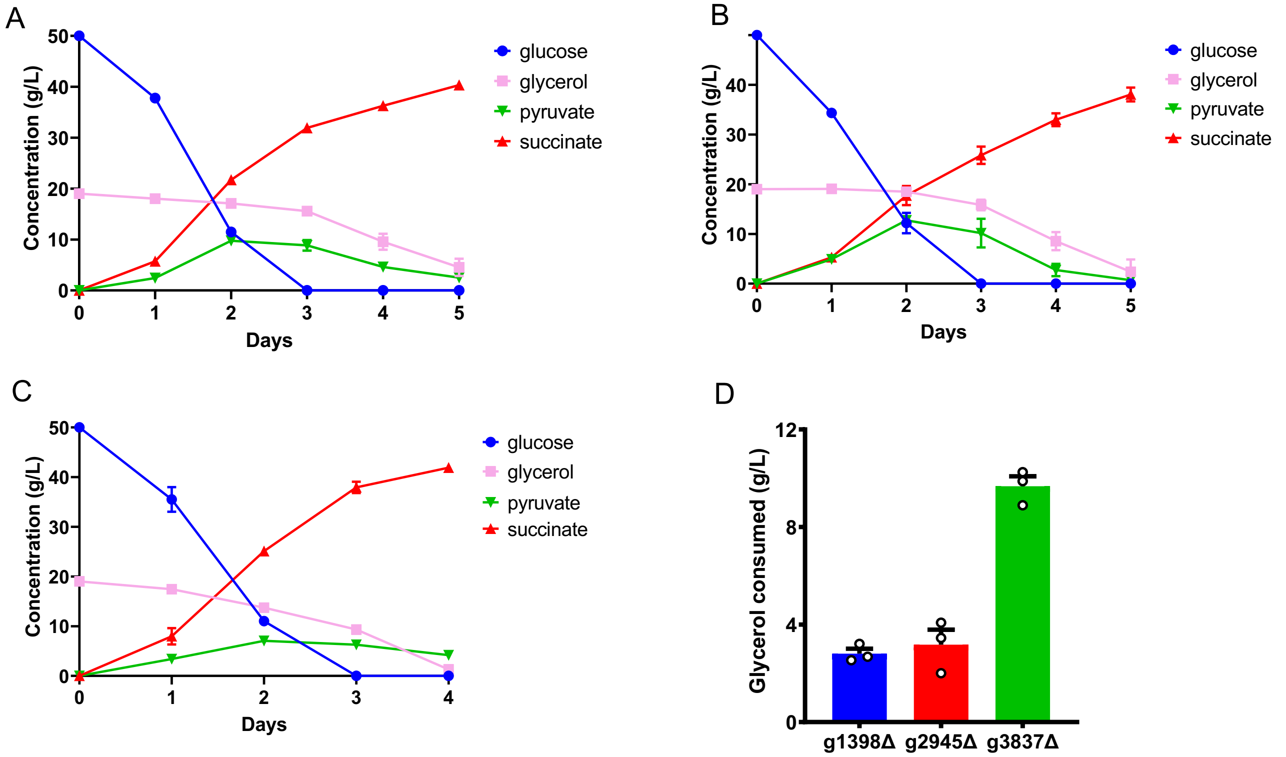

Supplementary Figure 9. Fermentation profiles in SC-URA medium with 50 g/L glucose and 20 g/L glycerol under aerobic condition in shake flasks. A. Strain g3473Δ/PaGDH-DAK with g1398 deletion. Error bars, mean ± SD (*n* = 3 biologically independent samples). **B**. Strain g3473Δ/PaGDH-DAK with g2945 deletion. Error bars, mean ± SD (*n* = 3 biologically independent samples). **C**. Strain g3473Δ/PaGDH-DAK with g3837 deletion. Error bars, mean ± SD (*n* = 3 biologically independent samples). **D**. Quantification of the amount of glycerol consumed when glucose was depleted. Error bars, mean ± SD (*n* = 3 biologically independent samples). Source data are provided as a Source Data file.

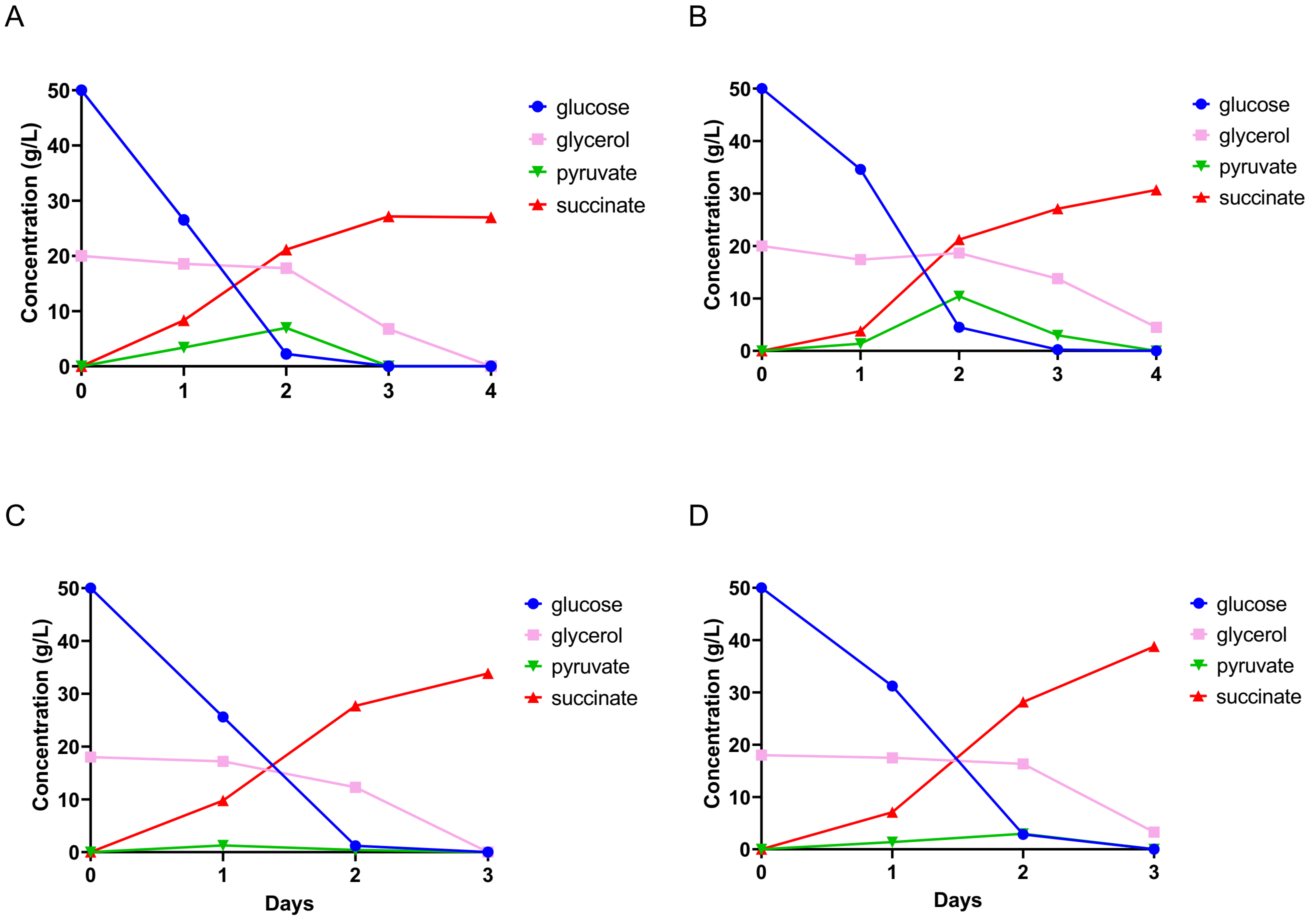

Supplementary Figure 10. Fermentation profiles in SC-URA medium with 50 g/L glucose and 20 g/L glycerol in bioreactors. **A**. Strain g3473Δ/PaGDH-DAK at 0.167 vvm O_2 and 0.333 vvm CO_2. **B**. Strain g3473Δ/PaGDH-DAK at 0.167 vvm O_2 and 0.667 vvm CO_2. **C**. Strain g3473Δ/PaGDH-DAK/g3837Δ at 0.167 vvm O_2 and 0.333 vvm CO_2. **D**. Strain g3473Δ/PaGDH-DAK/g3837Δ at 0.167 vvm O_2 and 0.667 vvm CO_2. Source data are provided as a Source Data file.

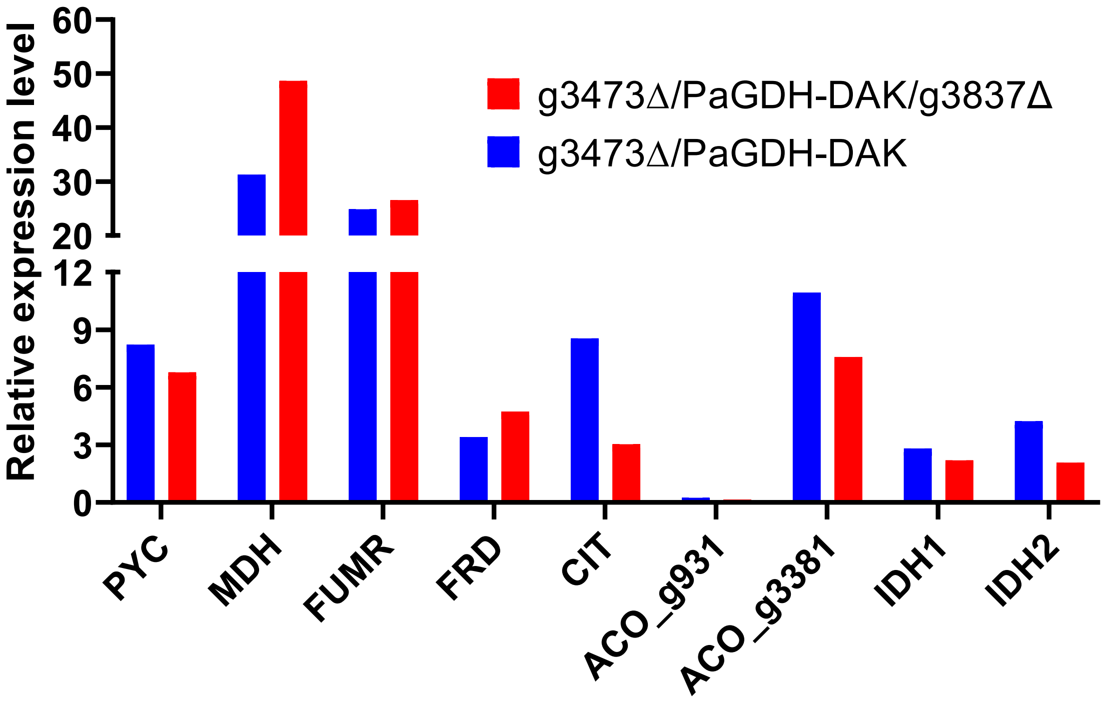

Supplementary Figure 11. qPCR analysis. Analysis of expression levels of *PYC*, *MDH*, *FUMR*, *FRD*, *CIT* (citrate synthase), g931 and g3381 (aconitase), and *IDH1* and *IDH2* (isocitrate dehydrogenase) compared to *alg9* in strains g3473Δ/PaGDH-DAK and g3473Δ/PaGDH-DAK/g3837Δ. Cells were grown in YP medium with glycerol as carbon source. Source data are provided as a Source Data file.

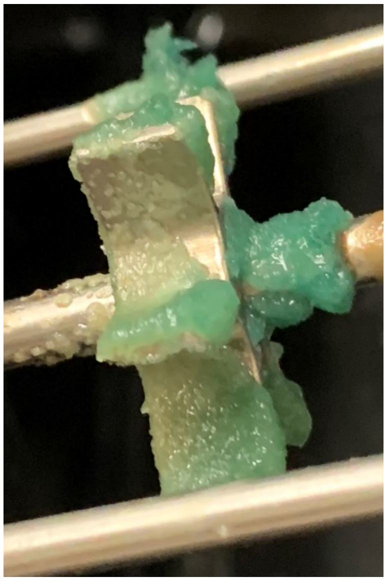

Supplementary Figure 12. Crystals after fed-batch fermentation using SC-URA medium.

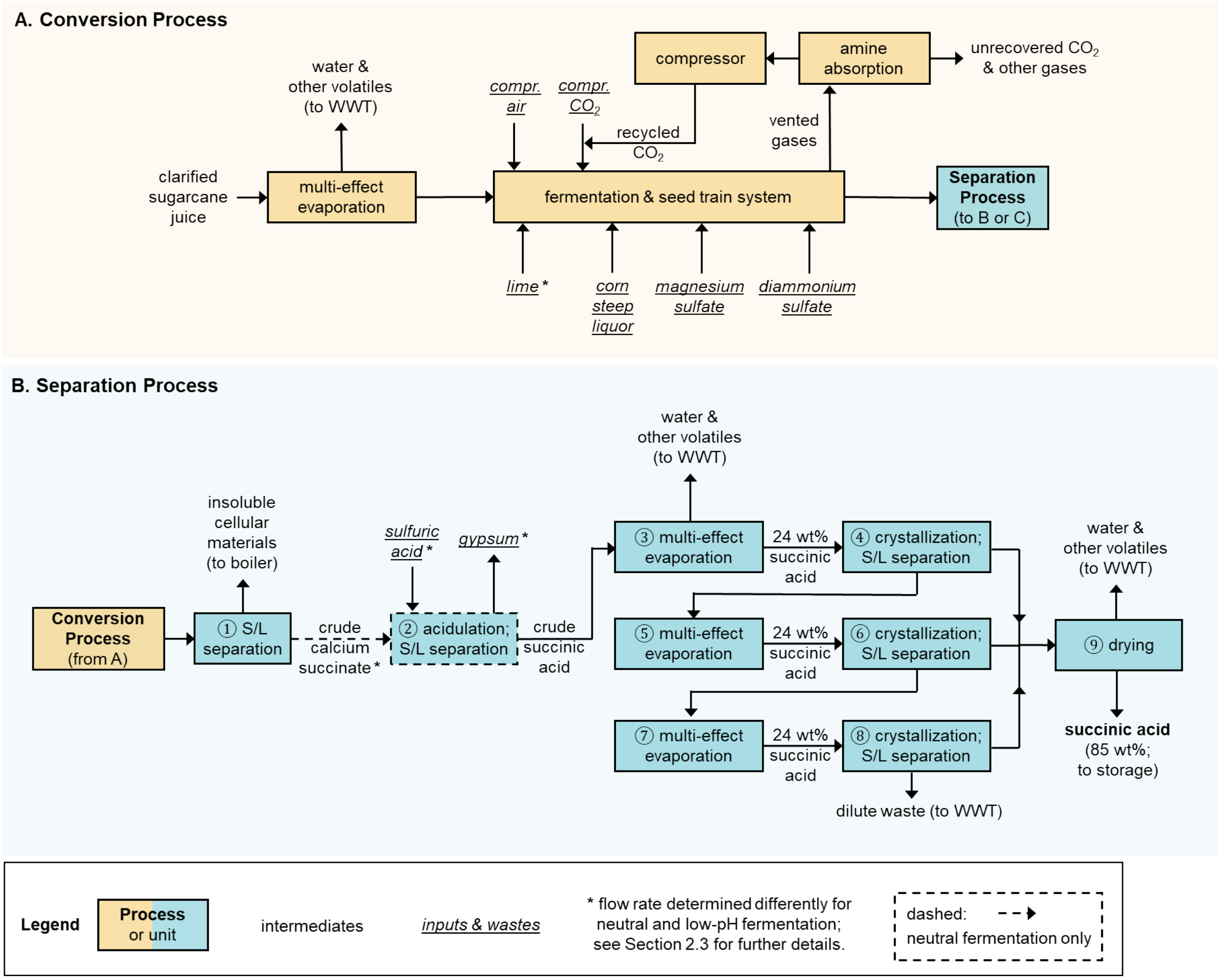

Supplementary Figure 13. Simplified block flow diagram of the designed biorefinery. A. Conversion process. **B**. Separation process. Acronyms denote compressed gas (compr.), wastewater treatment (WWT), and solid-liquid separation (S/L separation). A full process flowsheet is available online[19].

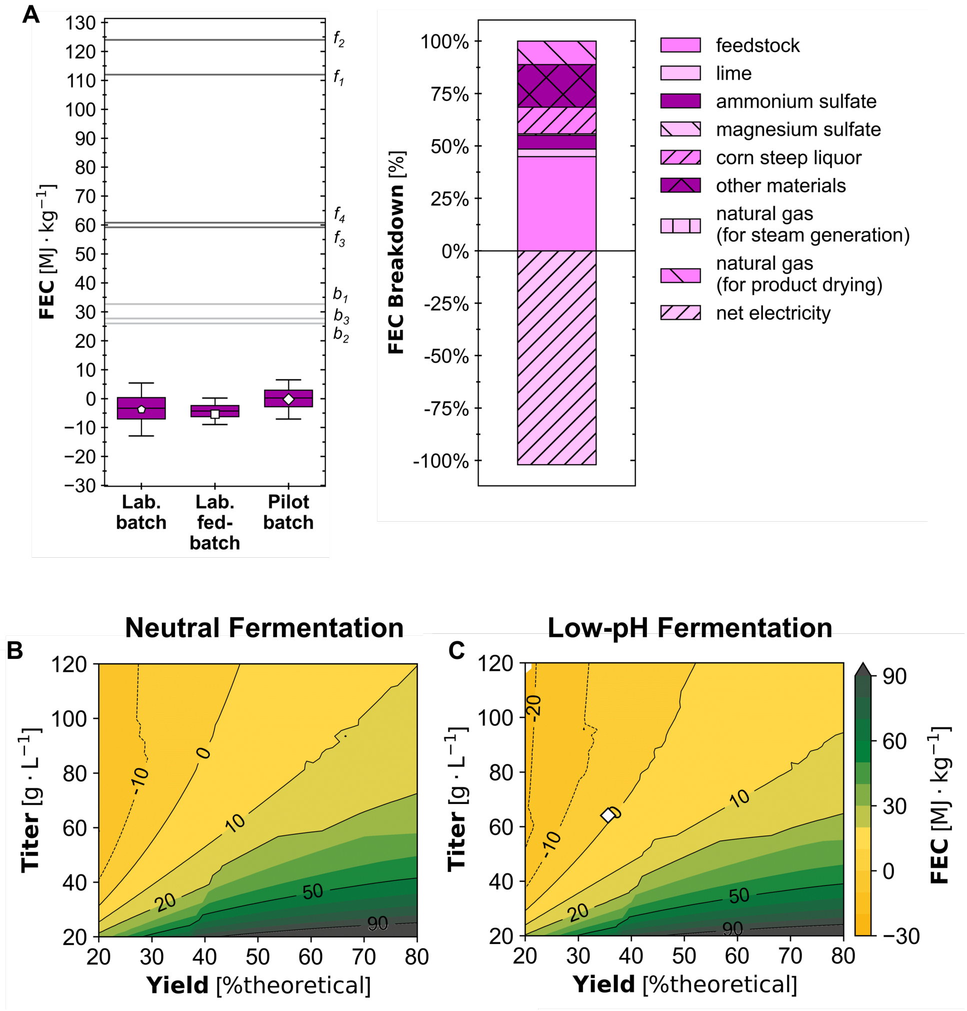

Supplementary Figure 14. FEC of the succinic acid biorefinery and effect of pH on FEC. A. Uncertainties (box and whisker plots) and breakdowns (stacked bar charts) for fossil energy consumption (FEC). Whiskers, boxes, and the middle line represent 5th/95th, 25th/75th, and 50th percentiles from 2,000 Monte Carlo simulations (n = 2000 simulations) for each scenario. Pentagon, square, and diamond markers represent baseline results for the *laboratory batch* (Lab. batch) *laboratory fed-batch* (Lab. fed-batch), and *pilot batch* (Pilot batch) *scenarios*, respectively. Stacked bar charts report baseline results for the *pilot batch scenario*; results for other scenarios are included in the SI. Electricity consumption includes only the consumption of the system; production was excluded in the depicted breakdown for figure clarity. Tabulated breakdown data for FEC are available online[19]. Labeled dark gray lines denote reported impacts for fossil-based production pathways (f1[16]; f2-f4[17]). Labeled light gray lines denote reported impacts for alternative bio-based production pathways (b1[17]; b2[18]; b3[10]). **B.** FEC across 2,500 fermentation yield-titer combinations at the baseline productivity of the *pilot batch scenario* (0.66 g/L/h) for neutral fermentation. **C**. FEC across 2,500 fermentation yield-titer combinations at the baseline productivity of the *pilot batch scenario* (0.66 g/L/h) for low-pH fermentation. Yield is shown as % of the theoretical maximum (%theoretical) scaled to the theoretical maximum yield of 1.31 g/g-glucose-equivalent (based on carbon balance). For a given point on the figure, the x-axis value represents the yield, the y-axis value represents the titer, and the color and contour lines represent the value of FEC. Diamond markers show baseline results for the *pilot batch scenario*. Source data are provided as a Source Data file.

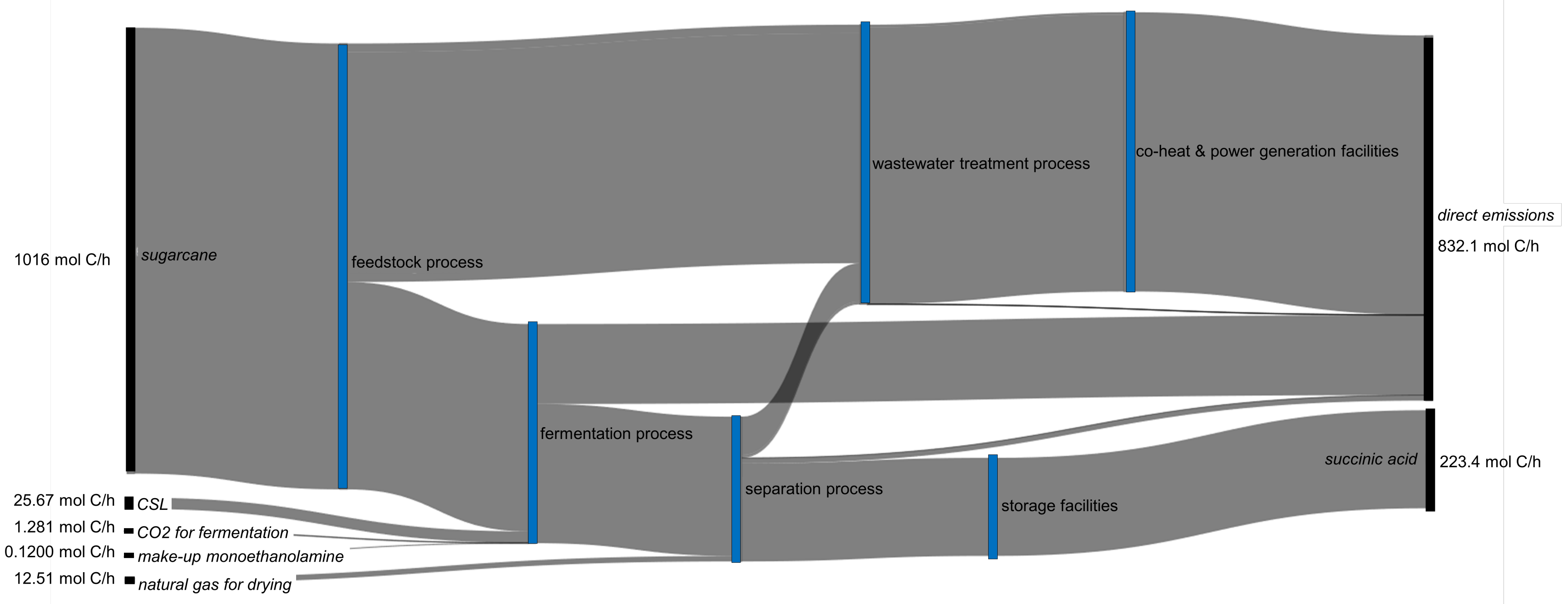

Supplementary Figure 15. Sankey diagram depicting the flow of carbon through the biorefinery for the *pilot batch scenario*. Italicized labels next to vertical black bars indicate biorefinery input and output streams containing carbon, a nd the values in mol C/h indicate the hourly molar flow of carbon in those streams. Non-italicized labels next to vertical blue bars indicate the biorefinery's processes and facilities. The vertical length of the gray Sankey flows indicates the magnitude of carbon flow input to, within, and output from the biorefinery. Source data are provided as a Source Data file.

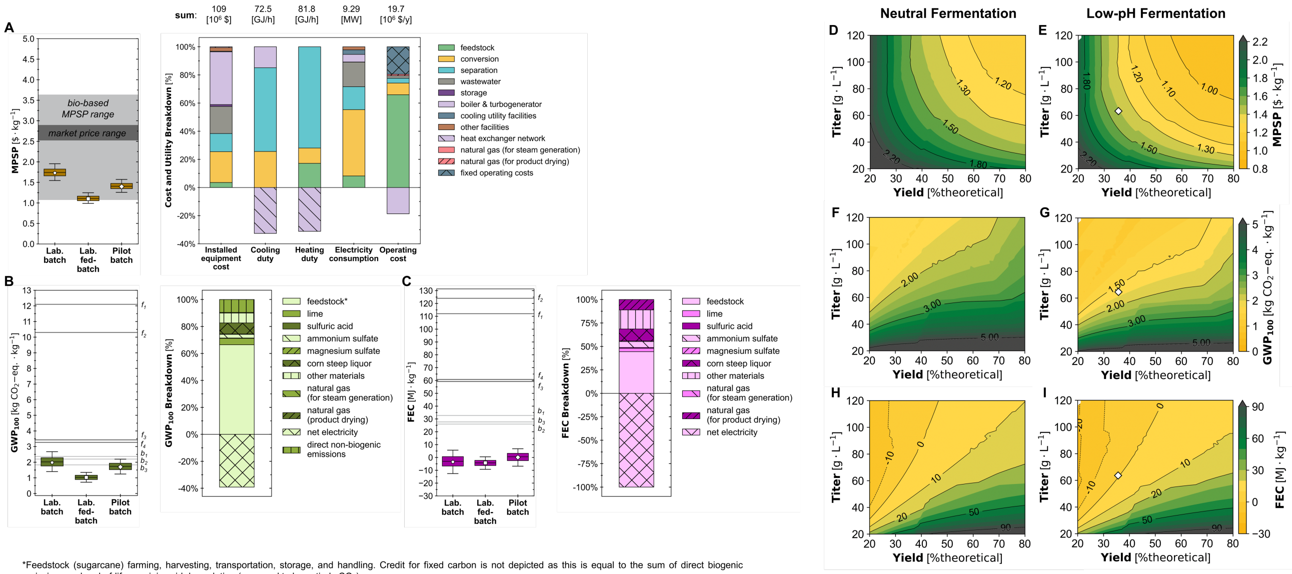

*Feedstock (sugarcane) farming, harvesting, transportation, storage, and handling. Credit for fixed carbon is not depicted as this is equal to the sum of direct biogenic emissions and end-of-life succinic acid degradation (assumed to be entirely CO_2).

Supplementary Figure 16. TEA/LCA results for an alternative low-pH scenario with re-acidulation required after fermentation. Uncertainties (box and whisker plots) and breakdowns (stacked bar charts) for (**A**) minimum product selling price (MPSP), (**B**) cradle-to-grave 100-year global warming potential (GWP_{100}), and (**C**) cradle-to-gate fossil energy consumption (FEC). Whiskers, boxes, and the middle line represent 5th/95th, 25th/75th, and 50th percentiles from 2,000 Monte Carlo simulations (n = 2000 simulations) for each scenario. Pentagon, square, and diamond markers represent baseline results for the *laboratory batch* (Lab. batch) *laboratory fed-batch* (Lab. fed-batch), and *pilot batch* (Pilot batch) *scenarios*, respectively. Stacked bar charts report baseline results for the *pilot batch scenario*; results for other scenarios are included in the SI. Tabulated breakdown data for material and installed equipment costs, heating and cooling duties, electricity usage, GWP_{100}, and FEC are available online[19]. Labeled dark gray lines denote reported impacts for fossil-based production pathways (f1[16]; f2-f4[17]). Labeled light gray lines denote reported impacts for alternative bio-based production pathways (b1[17]; b2[18]; b3[10]). Where GWP_{100} was reported as cradle-to-gate, 1.49 kg CO2-eq./kg was added as end-of-life impacts for consistency with this study and Dunn et al. 2015. Values for all reported MPSPs and impacts before and after adjustment are listed in Supplementary Table 3 and 4. (**D**, **E**) MPSP and (**F**, **G**) GWP_{100}, and (**H**, **I**) FEC across 2,500 fermentation yield-titer combinations at the baseline productivity of the *pilot batch scenario* (0.66 g/L/h) for neutral (left panel; **D**, **F**, **H**) and low-pH (right panel; **E**, **G**, **I**) fermentation. Yield is shown as % of the theoretical maximum (%theoretical) scaled to the theoretical maximum yield of 1.31 g/g-glucose-equivalent (based on carbon balance). For a given point on the figure, the x-axis value represents the yield, the y-axis value represents the titer, and the color and contour lines represent the value of MPSP, GWP_{100}. Diamond markers show baseline results for the *pilot batch scenario*. These results differ from those presented in **Figure 4** of the manuscript and **Supplementary Figure 13** in the SI as here, acidulation and S/L separation for gypsum removal was included for the low-pH scenario (the amount of sulfuric acid added for acidulation is assumed equivalent to the amount of lime added during fermentation for pH control). Source data are provided as a Source Data file.

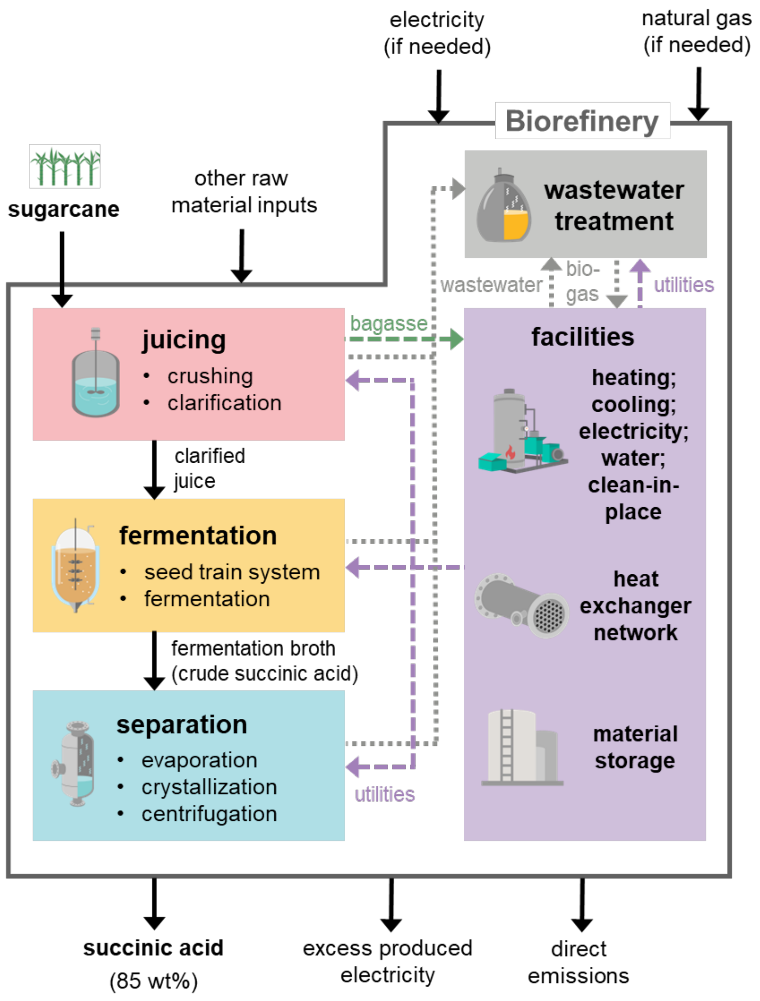

Supplementary Figure 17. Simplified overview of the biorefinery's process areas and input and output streams and utilities. The gray line labeled 'Biorefinery' denotes the system boundary.

Supplementary references

1. Shao, Z., Zhao, H. & Zhao, H. DNA assembler, an *in vivo* genetic method for rapid construction of biochemical pathways. *Nucleic Acids Res.* **37**, e16 (2009).

2. Engler, C., Kandzia, R. & Marillonnet, S. A one pot, one step, precision cloning method with high throughput capability. *PloS One* **3**, e3647 (2008).

3. Bates, R. L., Fondy, P. L., & Corpstein, R. R. Examination of some geometric parameters of impeller power. *Ind. Eng. Chem. Process Des. Dev*., **2**, 310-314 (1963).

4. Bhagwat, S. S. *et al.* Sustainable production of acrylic acid via 3-hydroxypropionic acid from lignocellulosic biomass. *ACS Sustain. Chem. Eng.* **9**, 16659–16669 (2021).

5. Xiao, H., Shao, Z., Jiang, Y., Dole, S. & Zhao, H. Exploiting *Issatchenkia orientalis* SD108 for succinic acid production. *Microb. Cell Fact.* **13**, 1-11 (2014).

6. Tran, V.G, Cao, M., Fatma, Z., Song, X. & Zhao, H. Development of a CRISPR/Cas9-based tool for gene deletion in *Issatchenkia orientalis*. *mSphere* **4**, e00345–19 (2019).

7. Ladakis, D., Stylianou, E., Ioannidou, S.-M., Koutinas, A. & Pateraki, C. Biorefinery development, techno-economic evaluation and environmental impact analysis for the conversion of the organic fraction of municipal solid waste into succinic acid and value-added fractions. *Bioresour. Technol.* **354**, 127172 (2022).

8. Ioannidou, S. M., Filippi, K., Kookos, I. K., Koutinas, A. & Ladakis, D. Techno-economic evaluation and life cycle assessment of a biorefinery using winery waste streams for the production of succinic acid and value-added co-products. *Bioresour. Technol.* **348**, 126295 (2022).

9. Shaji, A., Shastri, Y., Kumar, V., Ranade, V. V. & Hindle, N. Economic and environmental assessment of succinic acid production from sugarcane bagasse. *ACS Sustain. Chem. Eng.* **9**, 12738–12746 (2021).

10. Dickson, R. *et al.* Sustainable bio-succinic acid production: superstructure optimization, techno-economic, and lifecycle assessment. *Energy Environ. Sci.* **14**, 3542–3558 (2021).

11. Dogbe, E. S., Mandegari, M. & Görgens, J. F. Revitalizing the sugarcane industry by adding value to A-molasses in biorefineries. *Biofuels. Bioprod. Bioref.* **14**, 1089–1104 (2020).

12. Padi, R. K. & Chimphango, A. Feasibility of commercial waste biorefineries for cassava starch industries: Techno-economic assessment. *Bioresour. Technol.* **297**, 122461 (2020).

13. Klein, B. C. *et al.* Process development and techno-economic analysis of bio-based succinic acid derived from pentoses integrated to a sugarcane biorefinery. *Biofuels. Bioprod. Bioref.* **11**, 1051–1064 (2017).

14. Efe, Ç., van der Wielen, L. A. M. & Straathof, A. J. J. Techno-economic analysis of succinic acid production using adsorption from fermentation medium. *Biomass Bioenergy* **56**, 479–492 (2013).

15. Claypool, J. T. & Raman, D. R. Development and validation of a technoeconomic analysis tool for early-stage evaluation of bio-based chemical production processes. *Bioresour. Technol.* **150**, 486–495 (2013).

16. Dunn, J. B. *et al. Life-cycle Analysis of Bioproducts and Their Conventional Counterparts in GREET.* https://www.osti.gov/biblio/1250468/ (2015) doi:10.2172/1250468.

17. Cok, B., Tsiropoulos, I., Roes, A. L. & Patel, M. K. Succinic acid production derived from carbohydrates: An energy and greenhouse gas assessment of a platform chemical toward a bio-based economy. *Biofuels, Bioprod. Bioref.* **8**, 16–29 (2014).

18. Argonne National Laboratory. GREET 2022 Model. https://greet.anl.gov/ (2022).

19. BioSTEAM Development Group. Succinic acid sugarcane-based biorefinery (2023). URL: https://github.com/BioSTEAMDevelopmentGroup/Bioindustrial-Park/tree/master/biorefineries/succinic.
