# Supplementary material for: An end-to-end pipeline for succinic acid production at an industrially relevant scale using Issatchenkia orientalis
Source: Nat Commun. 2023 Oct 3;14:6152. doi: 10.1038/s41467-023-41616-9 (PMC10547785; doi:10.1038/s41467-023-41616-9)
Supplement: Supplementary file 3 — Description of Additional Supplementary Files [file 41467_2023_41616_MOESM3_ESM.pdf]

Description of Additional Supplementary Files

Supplementary Data 1: Details of parameters included in the uncertainty analysis. For neutral fermentation, the base required: succinic acid produced is assumed to be constant at 2 mol-OH-eq./mol, and the sulfuric acid requirement for downstream acidulation is assumed to be 2-mol-H+-eq./mol-succinic-acid.

Supplementary Data 2: Primers used in this study.

Supplementary Data 3: The customized core atom mapping model with redox balance and raw flux data.

Supplementary Data 4: The DNA sequences of all plasmids used in this study.
