# Supplementary material for: An end-to-end pipeline for succinic acid production at an industrially relevant scale using Issatchenkia orientalis
Source: Nat Commun. 2023 Oct 3;14:6152. doi: 10.1038/s41467-023-41616-9 (PMC10547785; doi:10.1038/s41467-023-41616-9)
Supplement: Supplementary file 8 — Reporting Summary [file 41467_2023_41616_MOESM8_ESM.pdf]

nature portfolio

Corresponding author(s): Huimin Zhao, Vijay Singh, Jeremy Guest

Last updated by author(s): Aug 29, 2023

Reporting Summary

Nature Portfolio wishes to improve the reproducibility of the work that we publish. This form provides structure for consistency and transparency in reporting. For further information on Nature Portfolio policies, see our Editorial Policies and the Editorial Policy Checklist.

Statistics

For all statistical analyses, confirm that the following items are present in the figure legend, table legend, main text, or Methods section.

n/a	Confirmed	
☐	☒	The exact sample size (*n*) for each experimental group/condition, given as a discrete number and unit of measurement
☐	☒	A statement on whether measurements were taken from distinct samples or whether the same sample was measured repeatedly
☐	☒	The statistical test(s) used AND whether they are one- or two-sided<br>*Only common tests should be described solely by name; describe more complex techniques in the Methods section.*
☒	☐	A description of all covariates tested
☒	☐	A description of any assumptions or corrections, such as tests of normality and adjustment for multiple comparisons
☐	☒	A full description of the statistical parameters including central tendency (e.g. means) or other basic estimates (e.g. regression coefficient) AND variation (e.g. standard deviation) or associated estimates of uncertainty (e.g. confidence intervals)
☐	☒	For null hypothesis testing, the test statistic (e.g. *F*, *t*, *r*) with confidence intervals, effect sizes, degrees of freedom and *P* value noted<br>*Give P values as exact values whenever suitable.*
☒	☐	For Bayesian analysis, information on the choice of priors and Markov chain Monte Carlo settings
☒	☐	For hierarchical and complex designs, identification of the appropriate level for tests and full reporting of outcomes
☒	☐	Estimates of effect sizes (e.g. Cohen's *d*, Pearson's *r*), indicating how they were calculated

Our web collection on statistics for biologists contains articles on many of the points above.

Software and code

Policy information about availability of computer code

Data collection	Data for the performance of simulated technologies was obtained from experimental results (for fermentation and crystallization) and BioSTEAM. Full reports for all simulations may be viewed in the online repository. GitHub repository with all code used for biorefinery modeling, simulation, techno-economic analysis, and life cycle assessment: https://github.com/BioSTEAMDevelopmentGroup/Bioindustrial-Park/tree/master/biorefineries/succinic
Data analysis	BioSTEAM and other custom Python scripts (open-source) were used to perform techno-economic analysis and life cycle assessment under uncertainty and across design and technological landscapes.

For manuscripts utilizing custom algorithms or software that are central to the research but not yet described in published literature, software must be made available to editors and reviewers. We strongly encourage code deposition in a community repository (e.g. GitHub). See the Nature Portfolio guidelines for submitting code & software for further information.

Data

Policy information about availability of data

All manuscripts must include a data availability statement. This statement should provide the following information, where applicable:

- Accession codes, unique identifiers, or web links for publicly available datasets
- A description of any restrictions on data availability
- For clinical datasets or third party data, please ensure that the statement adheres to our policy

The data supporting the findings of this work are available within the paper and its Supplementary Information files or uploaded through public repositories. If

specific data is believed to be missing, that data is available from the corresponding authors upon request. The source data underlying Figures 2-4, S1-S11, S14-S16, and Table S10 are provided as a Source Data file. The plasmids will be available through Addgene.

Research involving human participants, their data, or biological material

Policy information about studies with human participants or human data. See also policy information about sex, gender (identity/presentation), and sexual orientation and race, ethnicity and racism.

Reporting on sex and gender	Not applicable for this study.
Reporting on race, ethnicity, or other socially relevant groupings	Not applicable for this study.
Population characteristics	Not applicable for this study.
Recruitment	Not applicable for this study.
Ethics oversight	Not applicable for this study.

Note that full information on the approval of the study protocol must also be provided in the manuscript.

Field-specific reporting

Please select the one below that is the best fit for your research. If you are not sure, read the appropriate sections before making your selection.

☒ Life sciences ☐ Behavioural & social sciences ☐ Ecological, evolutionary & environmental sciences

For a reference copy of the document with all sections, see nature.com/documents/nr-reporting-summary-flat.pdf

Life sciences study design

All studies must disclose on these points even when the disclosure is negative.

Sample size	No statistical methods were used to predetermine sample size. All experiments, except for pilot-scale fermentation, were performed with at least two biological replicates; the results obtained suggested the chosen sample sizes were appropriate because clear distinctions could be seen with the current sample size. For pilot-scale fermentation, the data reported was obtained from a single replicate due to materials, volume, and budget considerations.
Data exclusions	No data was excluded from analysis. All the experimental/analysis results are included in the manuscript or Supplementary Information.
Replication	Fermentations using shake flasks were performed with biological triplicates. Metabolic flux analysis were performed with biological triplicates. qPCR analysis was performed with biological duplicates. Fermentations in bench-top reactors were performed with biological duplicates. Fermentations in pilot-scale reactors was performed with a single replicate. Succinic acid recovery, crystallization, and purification were performed with biological duplicates. All attempts at replication were successful.
Randomization	No randomization was performed as none of the experiments involved allocation of samples to test groups.
Blinding	Not relevant because no group allocation was involved in this study.

Reporting for specific materials, systems and methods

We require information from authors about some types of materials, experimental systems and methods used in many studies. Here, indicate whether each material, system or method listed is relevant to your study. If you are not sure if a list item applies to your research, read the appropriate section before selecting a response.

Materials & experimental systems

n/a	Involved in the study	
☒	☐	Antibodies
☒	☐	Eukaryotic cell lines
☒	☐	Palaeontology and archaeology
☒	☐	Animals and other organisms
☒	☐	Clinical data
☒	☐	Dual use research of concern
☒	☐	Plants

Methods

n/a	Involved in the study	
☒	☐	ChIP-seq
☒	☐	Flow cytometry
☒	☐	MRI-based neuroimaging
